# An asymptotic formula for the variance of the number of zeroes of a stationary Gaussian process

**DOI:** 10.1007/s00440-023-01218-4

**Published:** 2023-09-23

**Authors:** Eran Assaf, Jeremiah Buckley, Naomi Feldheim

**Affiliations:** 1 Dartmouth College, Hanover, NH USA; 2 King’s College, London, UK; 3 Bar-Ilan University, Ramat-Gan, Israel

**Keywords:** Gaussian process, Stationary process, Fluctuations of zeroes, Wiener Chaos, Primary: 60G10, 60G15, Secondary: 05A19, 37A46, 42A38

## Abstract

We study the variance of the number of zeroes of a stationary Gaussian process on a long interval. We give a simple asymptotic description under mild mixing conditions. This allows us to characterise minimal and maximal growth. We show that a small (symmetrised) atom in the spectral measure at a special frequency does not affect the asymptotic growth of the variance, while an atom at any other frequency results in maximal growth.

## Introduction

Zeroes of Gaussian processes, and in particular stationary Gaussian processes (SGPs), have been widely studied, with diverse applications in physics and signal processing; for a comprehensive historical account see [19]. The expected number of zeroes may be computed by the celebrated Kac–Rice formula. Estimating the fluctuations, however, proved to be a much more difficult task. The aim of this paper is to give a simple expression which describes the growth of the variance of the number of zeroes in the interval [0, *T*], as $T\rightarrow \infty$. Following the ideas of Slud [29], it is easy to give a lower bound for this quantity. Our main contribution is a matching upper bound, which holds under a very mild hypothesis. In particular we give a sharp asymptotic expression for the variance for any process with decaying correlations, no matter how slow the decay.

An intriguing feature of our results is the emergence of a ‘special frequency’: adding an atom to the spectral measure at this frequency does not change the order of growth of the fluctuations.

### Results

Let $f:\mathbb {R}\rightarrow \mathbb {R}$ be a stationary Gaussian process (SGP) with continuous *covariance kernel*

$$\begin{aligned} r(t) = {\mathbb {E}}[f(0) f(t)]. \end{aligned}$$

Denote by $\rho$ the *spectral measure* of the process, that is, the unique finite, symmetric measure on $\mathbb {R}$ such that

$$\begin{aligned} r(t) = \mathcal {F}[\rho ](t)= \int _\mathbb {R}e^{-i \lambda t} d\rho (\lambda ). \end{aligned}$$

We normalise the process so that $r(0) =\rho (\mathbb {R})= 1$. It is well-known (see, e.g., [7, Section 7.6]) that the distribution of *f* is determined by $\rho$, and further that any such $\rho$ is the spectral measure of some SGP.

We study the number of zeroes of *f* in a long ‘time’ interval [0, *T*], which we denote

$$\begin{aligned} N(\rho ;T) = N(T) =\# \{ t\in [0,T]: \, f(t)=0\}. \end{aligned}$$

The expectation of *N*(*T*) is given by the Kac–Rice formula (see [13, 32])

1 $$\begin{aligned} {\mathbb {E}}[N(T)] = \frac{\sigma }{\pi } T, \end{aligned}$$

where

$$\begin{aligned} \sigma ^2 = -r''(0) = \int _\mathbb {R}\lambda ^2 d\rho (\lambda ). \end{aligned}$$

Throughout we assume that *N*(*T*) has finite variance, which turns out to be equivalent to the *Geman condition* [11]

2 $$\begin{aligned} \int _0^{\varepsilon } \frac{r''(t) - r''(0) }{t} dt < \infty \quad \text {for some}\quad \varepsilon >0. \end{aligned}$$

An SGP *f* is *degenerate* if its spectral measure consists of a single symmetrised atom $\rho = \delta ^*_\alpha = \frac{1}{2} (\delta _{\alpha } + \delta _{-\alpha })$, or equivalently if the covariance is $r(t) = \cos (\alpha t)$. In this case the zero set is a random shift of the lattice $\frac{\pi }{\alpha }{\mathbb {Z}}$, and the variance ${{\,\textrm{Var}\,}}[N(T)]$ is bounded. Throughout this paper, atoms in the spectral measure should always be understood as symmetrised atoms.

We formulate our results in terms of the function

3 $$\begin{aligned} \varphi (t) = \max \left\{ |r(t)| + \frac{|r'(t)|}{\sigma }, \, \frac{|r'(t)|}{\sigma } + \frac{|r''(t)|}{\sigma ^2} \right\} . \end{aligned}$$

We note that^1^ the condition $r(t)\overset{|t|\rightarrow \infty }{\longrightarrow }\ 0$ implies that $\varphi (t)\overset{|t|\rightarrow \infty }{\longrightarrow }0$. This means that the condition (4) below may be viewed as a very mild mixing condition, which in particular holds whenever the spectral measure is absolutely continuous.

The notation $A(T) \asymp B(T)$ denotes that there exist $C_1, C_2>0$ such that $C_1 \le \frac{A(T)}{B(T)}\le C_2$ for all $T>0$, while $A(T)\sim B(T)$ denotes that $\lim _{T\rightarrow \infty } \frac{A(T)}{B(T)} = 1$. Our main result is the following.

#### Theorem 1

- For any SGP satisfying
  4 $$\begin{aligned} \limsup _{|t|\rightarrow \infty } \varphi (t) <1, \end{aligned}$$
  we have
  5 $$\begin{aligned} {{\,\textrm{Var}\,}}[N(T)] \asymp T\int _{0}^T \left( 1-\frac{t}{T}\right) \left( r(t)+\frac{r''(t)}{\sigma ^2}\right) ^2 \ dt \end{aligned}$$
  where the implicit constants depend on $\rho$.
- Under the additional assumptions $r+\frac{r''}{\sigma ^2} \not \in \mathcal {L}^2(\mathbb {R})$ and $\lim _{|t|\rightarrow \infty } \varphi (t) = 0$ we have
  $$\begin{aligned} {{\,\textrm{Var}\,}}[N(T) ] \sim \frac{\sigma ^2}{\pi ^2} T\int _{0}^T \left( 1-\frac{t}{T}\right) \left( r(t)+\frac{r''(t)}{\sigma ^2}\right) ^2 dt. \end{aligned}$$
- ${{\,\textrm{Var}\,}}[N(T)] \asymp T^2$ if and only if $\rho$ contains an atom at a point different from $\sigma$.

Following the ideas of Kac–Rice, one may write down an exact expression for ${{\,\textrm{Var}\,}}[N(T)]$, see, e.g., [7, Sections 10.6-7] or [11, Page 979]. While one may obtain some asymptotics from this expression if *r* decays at infinity, in general there are cancellations which are difficult to see explicitly. The main point of Theorem 1 is that the dominant contribution to ${{\,\textrm{Var}\,}}[N(T)]$ comes from $(r+\frac{r''}{\sigma ^2})^2$, due to other contributions cancelling, and much of our proofs involve organising terms appropriately to see this cancellation. We do this by considering the Wiener chaos expansion—the second chaos (which is the first non-trivial chaos) is an obvious lower bound and we dedicate much effort to showing that it is also an upper bound (up to a constant), under the hypothesis (4). This is the key estimate which allows us to prove stronger results than those which were known previously. Our proof boils down to proving some combinatorial identities for the coefficients of certain polynomials, see Sect. 1.5 for more details.

We obtain the following characterisation of linear variance from the proof of Theorem 1.

#### Corollary 2

We have

$$\begin{aligned} {{\,\textrm{Var}\,}}[N(T)] \asymp T\implies r+ \frac{r''}{\sigma ^2} \in \mathcal {L}^2(\mathbb {R}). \end{aligned}$$

Under condition (4), the converse holds.

The idea of using the first (non-trivial) chaos to give a lower bound for the variance goes back to Slud [28], see Sect. 1.3 for a discussion of previous results. While we were preparing this paper we became aware of the independent work [22], where this idea also appears. In particular, it is shown that ${{\,\textrm{Var}\,}}[N(T)]$ always grows at least linearly in *T* and that $r+\frac{r''}{\sigma ^2}\in \mathcal {L}^2(\mathbb {R})$ is necessary for linear variance. Both of these results also follow from Proposition 11 below. In [22] a sufficient condition for linear variance is also given, which essentially amounts to the condition $r+\frac{r''}{\sigma ^2}\in \mathcal {L}^2(\mathbb {R})$ and the spectral measure having an $\mathcal {L}^2$ density in a neighbourhood of $\pm \sigma$. These imply that $r,r''\in \mathcal {L}^2(\mathbb {R})$ and so $\lim _{|t|\rightarrow \infty } \varphi (t) =0$. Corollary 2 is therefore a stronger result than [22, Theorem 2.1 (ii)]. For instance, Corollary 2 allows us to conclude that we have linear variance for the example given in Sect. 1.4 below. It also allows us to see that we still have linear variance if we perturb a process that has linear variance by adding an atom (that is not too big) at $\sigma$, à la Corollary 3 below. These examples could not be analysed previously and we emphasise that the key difference is our ability to prove an upper bound for the variance, which allows us to prove stronger results.

By stationarity, ${{\,\textrm{Var}\,}}[N(T) ]$ grows at most quadratically in *T* and so Theorem 1 (c) therefore characterises maximal growth. Again, one direction of this result also appeared in [22, Theorem 2.1 (iii)], but our results are stronger due to our upper bound.

The emergence of a special frequency $\sigma$ in Theorem 1 (c) is new,^2^ and intriguing. One naturally asks what the effect of an atom at this frequency is. Notice that modifying a measure by adding an atom at frequency $\sigma$ does not change ${\mathbb {E}}[N(T)]$. The following result follows from Theorem 1 (a), and shows that the asymptotic growth of ${{\,\textrm{Var}\,}}[N(T)]$ remains unchanged as well—at least under some mild assumptions.

#### Corollary 3

Suppose that (4) holds for the spectral measure $\rho$. Define^3^$\rho _\theta =(1-\theta ) \rho + \theta \delta ^*_\sigma$ for $0<\theta <1$. There exists $\theta _0>0$ such that

$$\begin{aligned} {{\,\textrm{Var}\,}}[N(\rho ; T)] \asymp {{\,\textrm{Var}\,}}[ N(\rho _\theta ; T)] \end{aligned}$$

for any $\theta <\theta _0$ (and the implicit constants may depend on $\theta$). Moreover, $\theta _0$ depends only on $\limsup _{|t| \rightarrow \infty } \varphi (t)$.

### Discussion

As we already remarked, a major theme of our results is the importance of the quantity $r + \frac{r''}{\sigma ^2}$, since we use it to give both upper and lower bounds for ${{\,\textrm{Var}\,}}[N(T)]$. Let us first note that there might also be cancellation within this expression, see Sect. 1.4 for an example of $r,r''\notin \mathcal {L}^2$ but $r + \frac{r''}{\sigma ^2}\in \mathcal {L}^2$.

Observe also that $r + \frac{r''}{\sigma ^2}=\mathcal {F}[\mu ]$ where the signed measure $\mu$ is defined by $d\mu (\lambda )=\left( 1 - \frac{\lambda ^2}{\sigma ^2} \right) d \rho (\lambda )$; this is crucial to some of our proofs. In fact, it follows from Parseval’s identity that

6 $$\begin{aligned} \int _{0}^T \left( 1-\frac{t}{T}\right) \left( r(t)+\frac{r''(t)}{\sigma ^2}\right) ^2 \ dt = \pi \int (\mathcal {S}_T * \mu )\ d\mu \end{aligned}$$

where $\mathcal {S}_T(\lambda ) = \frac{T}{2\pi } \ {{\,\textrm{sinc}\,}}^2\left( \frac{T \lambda }{2}\right) .$ For details, see Sect. 4.2. One consequence of the cancellation mentioned above is the emergence of the special atom (in the sense of Theorem 1 (c) and Corollary 3). This phenomenon is explained, in part, by the fact that the measure $\mu$ does not ‘see’ $\sigma$.

For crossings of non-zero levels, the presence of an atom at any frequency leads to quadratic variance, see the remark on Page 18 after the proof of Theorem 1 (c). The existence of a special atom at a distinguished frequency is therefore unique to the zero level. Furthermore, this phenomenon is purely real. No such frequency exists for complex zeroes, see [10].

We remark that, following Arcones [3], many previous results were stated in terms of the function

$$\begin{aligned} \psi (t) = \max \left\{ |r(t)|, \frac{|r'(t)|}{\sigma }, \frac{|r''(t)|}{\sigma ^2} \right\} \end{aligned}$$

rather than the function $\varphi$ that we introduced in (3). To compare the two, note that our assumption (4) is implied by the stronger assumption $\limsup _{|t|\rightarrow \infty } \psi (t) <\frac{1}{2}$.

While the condition (4) is a very mild mixing condition, there are some processes with singular spectral measure for which it does not hold. We believe that our results hold in greater generality.

#### Conjecture

(Weak form) The estimate (5) holds for any non-degenerate SGP satisfying

7 $$\begin{aligned} \limsup _{|t|\rightarrow \infty } \max \left\{ r(t)^2 + \frac{r'(t)^2}{\sigma ^2}, \, \frac{r''(t)^2}{\sigma ^4} + \frac{r'(t)^2}{\sigma ^2} \right\} <1. \end{aligned}$$

#### Conjecture

(Strong form) The estimate (5) holds for any non-degenerate SGP.

Even the weak form of the conjecture would allow us to prove stronger results, e.g., to prove that Corollary 3 holds for any $\theta \in [0,1)$. The strong form would allow us to improve Corollary 2 to completely characterise linear variance. We provide further evidence for the conjectures in Sect. 3.5. We also note that every SGP satisfies $\max \left\{ r(t)^2 + \frac{r'(t)^2}{\sigma ^2}, \, \frac{r''(t)^2}{\sigma ^4} + \frac{r'(t)^2}{\sigma ^2} \right\} \le 1$ and if equality holds for any finite $t\ne 0$ then the process is degenerate. The condition (7) is therefore extremely mild.

### Background and motivation

The origins for the Kac–Rice method for computing the expected number of zeroes lie in the independent work of Kac [15, 16] and of Rice [25, 26]. Applying this method to SGPs yields the formula (1), even when both sides are infinite, as was done by Ylvisaker [32] and Itô [13]. Sufficiency of the Geman condition (2) for finite variance was proved by Cramér and Leadbetter [7, Equation 10.6.2 or 10.7.5], while necessity was established by Geman [11]. Qualls [24, Lemma 1.3.4] showed that the Geman condition is equivalent to the spectral condition $\int _\mathbb {R}\log (1+|\lambda |)\lambda ^2 d\rho (\lambda )<\infty$ (see also [4, Theorem 3]).

An exact formula for the variance was rigorously derived^4^ by Cramér and Leadbetter [7, Sections 10.6-7], although extracting the rate of growth of the variance under general conditions from this expression proved challenging. Little progress in understanding the asymptotic growth of the variance was made until Slud [28, 29] introduced Multiple Wiener Integral techniques some decades later—these were in turn refined and extended by Kratz and Léon [20, 21], using Wiener chaos expansions. These formulas and techniques were used to prove various properties of the zeroes, such as sufficient conditions for linearity of the variance and for a central limit theorem (see, e.g., [8, 23]).

The case of linear variance was historically of interest. Previously, the only condition for asymptotically linear variance (that we are aware of) was $r,r''\in \mathcal {L}^2(\mathbb {R})$, which follows from combining the results of Cuzick [8] and Slud [28]. We show in Sect. 1.4 that the condition $r+ \frac{r''}{\sigma ^2} \in \mathcal {L}^2(\mathbb {R})$ is strictly weaker, therefore Corollary 2 improves upon their result. It also follows from their work that $r, r'' \in \mathcal {L}^2(\mathbb {R})$ implies that $\frac{1}{T}{{\,\textrm{Var}\,}}[N(T)]$ converges as $T\rightarrow \infty$. Ancona and Letendre [1, Proposition 1.11] give an exact expression for this limit (see also [9, Proposition 3.1]), although their main focus is on the growth of the central moments of linear statistics (which generalise the zero count). A linear lower bound appears in the (independent) work of Lachièze–Rey [22], who also studies rigidity and predictability of the zero set.

We finally mention that our work has parallels in different but related models. In the setting of complex zeroes of a random Gaussian analytic $f:{\mathbb {C}}\rightarrow {\mathbb {C}}$ an asymptotic formula for the variance, an $L^2$-condition that guarantees linearity, and a characterisation of maximal (i.e., quadratic) growth were given in [10]. Analogous results were then proved for the winding number of a Gaussian stationary $f:\mathbb {R}\rightarrow {\mathbb {C}}$ in [5].

### Cancellation in the quantity $r + \frac{r''}{\sigma ^2}$

As we indicated previously, an important message of this paper is that the behaviour of the variance is governed by the quantity $r + \frac{r''}{\sigma ^2}$. We wish to emphasise the important rôle of cancellation between the two terms here, and we have already seen an example of this in Corollary 3 when the spectral measure has an atom at a ‘special frequency’. However this cancellation phenomenon is not just about atoms, and as an illustrative example we will produce a^5^ covariance function *r* such that:

- The spectral measure $\rho$ has an $\mathcal {L}^1(\mathbb {R})$ density.
- $$r + \frac{r''}{\sigma ^2} \in \mathcal {L}^2(\mathbb {R})$$ where $$\sigma ^2 = \int _{\mathbb {R}} \lambda ^2 \ d\rho (\lambda )$$.
- $$r, r'' \notin \mathcal {L}^2(\mathbb {R})$$.

Writing $d\rho (\lambda )=\phi (\lambda )d\lambda$ and applying the Fourier transform we see that it is equivalent to produce a function $\phi \ge 0$ satisfying:

1. $$\int _{\mathbb {R}}\phi (\lambda ) d\lambda =1$$ but $$\phi \notin \mathcal {L}^2(\mathbb {R})$$.
2. $$\lambda ^2 \phi (\lambda ) \in \mathcal {L}^1(\mathbb {R})$$, but $$\lambda ^2 \phi (\lambda ) \notin \mathcal {L}^2(\mathbb {R})$$.
3. $$\left( 1 - \frac{\lambda ^2}{\sigma ^2} \right) \phi (\lambda ) \in \mathcal {L}^2(\mathbb {R})$$ where $$\sigma ^2 = \int _{\mathbb {R}} \lambda ^2 \phi (\lambda ) d\lambda $$.

We proceed to produce such a function $\phi$.

Let $\alpha \in \left( \frac{1}{2}, 1 \right)$. Choose $M > 1$ such that

8 $$\begin{aligned} M^2 + M + 1 > 3 + 3(1-\alpha ) \left( \frac{1}{3-\alpha } - \frac{2}{2-\alpha } \right) , \end{aligned}$$

and let $c_1, c_2 \in \mathbb {R}$ be the solution of the linear system

9 $$\begin{aligned} \begin{array}{ccccccc} \frac{1}{1-\alpha } &{} c_1 &{} + &{} (M-1) &{} c_2 &{} = &{} \frac{1}{2}, \\ \left( \frac{1}{1-\alpha } - \frac{2}{2-\alpha } + \frac{1}{3-\alpha } \right) &{} c_1 &{} + &{} \frac{M^3 - 1}{3} &{} c_2 &{} = &{} \frac{1}{2}. \end{array} \end{aligned}$$

We note that (8) ensures that the determinant of the matrix associated to (9) is positive, and since we also have $\frac{M^3 - 1}{3} > M - 1$ and $\frac{2}{2-\alpha } > \frac{1}{3-\alpha }$, it follows that $c_1, c_2 > 0$. Define

$$\begin{aligned} \phi (\lambda ) = {\left\{ \begin{array}{ll} c_1 (1 - |\lambda |)^{-\alpha }, &{} \text { for } |\lambda |< 1, \\ c_2, &{} \text { for } 1< |\lambda | < M. \end{array}\right. } \end{aligned}$$

Then:

- Since $\alpha \in \left( \frac{1}{2}, 1\right)$, it follows that $\phi \in \mathcal {L}^1(\mathbb {R})$ but $\phi \notin \mathcal {L}^2(\mathbb {R})$.
- Integration yields, by the first equation in (9), that $\int _{\mathbb {R}}\phi (\lambda ) d\lambda =1$.
- Similarly $$\lambda ^2 \phi (\lambda ) \in \mathcal {L}^1(\mathbb {R})$$, but $$\lambda ^2 \phi (\lambda ) \notin \mathcal {L}^2(\mathbb {R})$$.
- Now the second equation in (9) shows that $\sigma ^2 = \int _{\mathbb {R}} \lambda ^2 \phi (\lambda ) d\lambda = 1$.
- Finally note that $\left( 1 - \lambda ^2\right) \phi (\lambda ) \in \mathcal {L}^2(\mathbb {R})$.

### Outline of our methods

Let us briefly outline our method. We write

$$\begin{aligned} N(T)=\sum _{q=0}^\infty \pi _{q}(N(T)) \end{aligned}$$

where $\pi _q$ denotes the projection onto the *q*’th Wiener chaos. Explicit expressions for this decomposition are well known, it turns out that only the even chaoses contribute, and so we have

$$\begin{aligned} {{\,\textrm{Var}\,}}[N(T)]=\sum _{q=1}^\infty {\mathbb {E}}[\pi _{2q}(N(T))^2]. \end{aligned}$$

The diagram formula allows us to compute (see Lemma 5)

$$\begin{aligned} {\mathbb {E}}[\pi _{2q}(N(T))^2]=\int _{-T}^T (T-|t|){\widetilde{P}}_q(t) dt \end{aligned}$$

where ${\widetilde{P}}_q$ is a polynomial expression that involves $r,r'$ and $r''$. We establish that $\left( r+\frac{r''}{\sigma ^2}\right) ^2$ divides the polynomial^6^${\widetilde{P}}_q$ exactly, see Proposition 8. This yields

$$\begin{aligned} {\mathbb {E}}[\pi _{2q}(N(T))^2]\le C_q \int _{-T}^T (T-|t|) \left( r(t)+\frac{r''(t)}{\sigma ^2}\right) ^2 dt \end{aligned}$$

for some $C_q$. The remainder of our proof of the upper bound involves showing that this sequence $C_q$ is summable (in fact, decays exponentially) under the given hypothesis; we do this in Proposition 9. We finally remark that the fact that $\left( r+\frac{r''}{\sigma ^2}\right) ^2$ divides the polynomial ${\widetilde{P}}_q$ exactly seems like a miraculous coincidence, and it would be interesting to understand it better.

## A formula for the variance

The goal of this section is to give an infinite series expansion for ${{\,\textrm{Var}\,}}[N(T)]$, each coming from a different component of the Wiener chaos (or Hermite-Itô) expansion of *N*(*T*). We begin with some notation. For $q\in {\mathbb {N}}$ and $l, l_1, l_2, n \in {\mathbb {N}}_0 ={\mathbb {N}}\cup \{0\}$ write^7^

10 $$\begin{aligned} a_{q}(l) = \frac{1}{l! (q-l)!} \cdot \frac{1}{2l-1} \end{aligned}$$

and

11 $$\begin{aligned} b_{q}(l_{1},l_{2},n)=\frac{(2q-2l_{1})!(2l_{1})!(2q-2l_{2})!(2l_{2})!}{(2q-2l_{1}-2l_{2}+n)!(2l_{1}-n)!(2l_{2}-n)!n!}. \end{aligned}$$

Next define the polynomials

12 $$\begin{aligned} {\widetilde{P}}_{q}(x,y,z)= & {} \sum _{l_{1},l_{2}=0}^{q}a_{q}(l_{1}) a_q(l_{2})\nonumber \\ {}{} & {} \sum _{n=\max (0,2(l_{1}+l_{2}-q))}^{\min (2l_{1},2l_{2})} b_{q}(l_{1},l_{2},n)\cdot x^{2(q-l_{1}-l_{2})+n}y^{2(l_{1}+l_{2}-n)}z^{n} \end{aligned}$$

and

13 $$\begin{aligned} P_{q}(x,y,z) ={\widetilde{P}}_{q}(x,y,z) + c_{q}\left( x^{2q-1}z+(2q-1)x^{2q-2}y^{2}\right) \end{aligned}$$

where

14 $$\begin{aligned} c_{q}=\frac{2^{4q}(q!)^{2}}{2q(2q)!} = \frac{2^{4q}}{2q \left( {\begin{array}{c}2q\\ q\end{array}}\right) }. \end{aligned}$$

We are now ready to state the expansion.

### Proposition 4

We have

$$\begin{aligned} {{\,\textrm{Var}\,}}N(T) = \frac{\sigma ^2}{\pi ^2} \sum _{q=1}^{\infty } \frac{V_q(T)}{4^q} + \frac{\arccos r(T)}{\pi } \left( 1-\frac{\arccos r(T)}{\pi } \right) \end{aligned}$$

where

15 $$\begin{aligned} V_q(T) = 2\int _{0}^{T} \left( T - t \right) P_q \left( r(t), \frac{r'(t)}{\sigma }, \frac{r''(t)}{\sigma ^2} \right) dt. \end{aligned}$$

Furthermore

16 $$\begin{aligned} {{\,\textrm{Var}\,}}N(T) \ge \frac{\sigma ^2}{4\pi ^2} V_1(T) + \frac{1}{\pi ^2}\left( 1-r(T)^2 \right) . \end{aligned}$$

The starting point in our calculations is the following Hermite expansion for *N*(*T*) given by Kratz and Léon [21, Proposition 1] assuming only the Geman condition^8^ (though they and other authors had considered it previously under more restrictive assumptions). We have (the sum converges in $L^2({\mathbb {P}})$)

$$\begin{aligned} N(T) =\frac{\sigma }{\pi } \sum _{q=0}^\infty \frac{(-1)^{q+1}}{2^q} N_q(T) \end{aligned}$$

where.^9^

17 $$\begin{aligned} N_q(T) = \sum _{l=0}^q a_q(l)\int _0^T H_{2(q-l)}(f(t)) H_{2l} (f'(t)/\sigma ) \ dt, \end{aligned}$$

and $H_l$ is the *l*’th Hermite polynomial. Further each $N_q(T)$ belongs to the 2*q*’th Wiener chaos which yields

$$\begin{aligned} {\mathbb {E}}[N(T)] = \frac{\sigma }{\pi } N_0(T) = \frac{\sigma }{\pi }T, \end{aligned}$$

and

18 $$\begin{aligned} {{\,\textrm{Var}\,}}[N(T)] =\frac{\sigma ^2}{\pi ^2} \sum _{q=1}^\infty 4^{-q} {\mathbb {E}}[N_q(T)^2]. \end{aligned}$$

Furthermore

19 $$\begin{aligned} {{\,\textrm{Var}\,}}[N(T)]\ge \frac{\sigma ^2}{\pi ^2} \frac{{\mathbb {E}}[N_1(T)^2]}{4}. \end{aligned}$$

The next lemma allows us to evaluate ${\mathbb {E}}\left[ N_q(T)^2 \right]$

### Lemma 5

For all $$q \in {\mathbb {N}}$$

$$\begin{aligned} {\mathbb {E}}\left[ N_q(T)^2 \right] = 2\int _{0}^{T} \left( T - t \right) {\widetilde{P}}_q \left( r(t), \frac{r'(t)}{\sigma }, \frac{r''(t)}{\sigma ^2} \right) dt, \end{aligned}$$

where ${\widetilde{P}}_q$ is given by (12).

We now show how this lemma yields the desired expression.

### Proof of Proposition 4, assuming Lemma 5

Lemma 5 yields

$$\begin{aligned} {\mathbb {E}}\left[ N_q(T)^2 \right] \!=\! V_q(T) - \frac{2c_q}{\sigma ^2} \!\int _{0}^{T} \!\!\left( T \!- t \right) \left( r(t)^{2q-1} r''(t) \!+\! (2q\!-\!1) r(t)^{2q-2} r'(t)^2 \right) \ dt. \end{aligned}$$

Note that $r(t)^{2q-1} r''(t) + (2q-1) r(t)^{2q-2} r'(t)^2 = \frac{d^2}{dt^2} \left[ \frac{r(t)^{2q}}{2q} \right]$ and so

$$\begin{aligned} \int _{0}^{T} \left( T - t \right)&\Big ( r(t)^{2q-1} r''(t) + (2q-1) r(t)^{2q-2} r'(t)^2 \Big ) \ dt\\&= \frac{1}{2q} \int _{0}^{T} (T - t) \frac{d^2}{dt^2} \left[ r(t)^{2q}\right] \ dt\\&= \frac{1}{2q} \left[ (T-t) \cdot 2q \cdot r(t)^{2q-1} r'(t) \Big \vert _{t=0}^{T} + \int _{0}^{T} \frac{d}{dt} \left[ r(t)^{2q} \right] \ dt \right] \\&= \frac{1}{2q} \left[ r(T)^{2q} - 1 \right] . \end{aligned}$$

We therefore have

$$\begin{aligned} {\mathbb {E}}\left[ N_q(T)^2 \right] = V_q(T) + \frac{c_q}{q \sigma ^2} \left( 1 - r(T)^{2q} \right) . \end{aligned}$$

Applying (19) yields the desired lower bound

$$\begin{aligned} {{\,\textrm{Var}\,}}[N(T)]\ge \frac{\sigma ^2}{4\pi ^2}{\mathbb {E}}[N_1(T)^2]= \frac{\sigma ^2}{4\pi ^2}V_1(T)+\frac{1}{\pi ^2}(1-r(T)^2) \end{aligned}$$

while (18) gives

$$\begin{aligned} {{\,\textrm{Var}\,}}\left[ N(T) \right]&= \frac{\sigma ^2}{\pi ^2} \sum _{q=1}^{\infty } \frac{1}{4^q} \left[ V_q(T) + \frac{c_q}{q \sigma ^2} \left( 1 - r(T)^{2q} \right) \right] \\&= \frac{\sigma ^2}{\pi ^2} \sum _{q=1}^{\infty } \frac{V_q(T)}{4^q} + \frac{1}{2\pi ^2} \sum _{q=1}^{\infty } \frac{2^{2q} - (2r(T))^{2q}}{q^2 {\left( {\begin{array}{c}2q\\ q\end{array}}\right) }}. \end{aligned}$$

We identify the last series as

20 $$\begin{aligned} \arcsin ^2(x) = \frac{1}{2} \sum _{q=1}^\infty \frac{2^{2q}}{q^2 \left( {\begin{array}{c}2q\\ q\end{array}}\right) } x^{2q} \end{aligned}$$

for all $|x|\le 1$ implying that

$$\begin{aligned} {{\,\textrm{Var}\,}}[ N(T) ]&= \frac{\sigma ^2}{\pi ^2} \sum _{q=1}^{\infty } \frac{V_q(T)}{4^q} + \frac{\arcsin ^2(1) - \arcsin ^2(r(T)) }{\pi ^2} \\&= \frac{\sigma ^2}{\pi ^2} \sum _{q=1}^{\infty } \frac{V_q(T)}{4^q} + \frac{\arccos r(T)}{\pi } \left( 1-\frac{\arccos r(T)}{\pi } \right) , \end{aligned}$$

where the last equality follows from $\arccos (x) = \frac{\pi }{2} - \arcsin (x)$. $\square$

We now proceed to prove Lemma 5.

### Proof of Lemma 5

Squaring the expression for $N_q(T)$ given in (17) yields

$$\begin{aligned} N_q(T)^2= & {} \sum _{l_1, l_2=0}^q a_q(l_1) a_q(l_2) \int _0^T\int _{0}^{T} H_{2(q-l_1)}(f(t)) \\{} & {} H_{2(q-l_2)}(f(s)) H_{2l_1} \left( \frac{f'(t)}{\sigma } \right) H_{2l_2} \left( \frac{f'(s)}{\sigma } \right) ds \,dt. \end{aligned}$$

and so

$$\begin{aligned} {\mathbb {E}}\big [ N_q(T)^2 \big ]&=\sum _{l_1, l_2=0}^q a_q(l_1) a_q(l_2) \int _0^T\int _{0}^{T} \\&{\mathbb {E}}\left[ H_{2(q-l_1)}(f(t)) H_{2(q-l_2)}(f(s)) H_{2l_1} \left( \frac{f'(t)}{\sigma } \right) \right. \\&\left. H_{2l_2}\left( \frac{f'(s)}{\sigma } \right) \right] ds\, dt. \end{aligned}$$

Applying Lemma 6 below, and using the simple change of variables

$$\begin{aligned} \int _0^T \int _0^T h(t-s) dt \, ds = \int _{-T}^T (T-|x|) h(x) dx \end{aligned}$$

for any $h\in L^1([-T,T])$, we get

$$\begin{aligned} {\mathbb {E}}\big [ N_q(T)^2 \big ]=\int _{-T}^{T} \left( T - |t| \right) {\widetilde{P}}_q \left( r(t), \frac{r'(t)}{\sigma }, \frac{r''(t)}{\sigma ^2} \right) dt. \end{aligned}$$

Noting that *r* is an even function and that only even powers of *y* appear in ${\widetilde{P}}_q$ yields Lemma 5. $\square$

### Lemma 6

For all $q\in {\mathbb {N}}$ and $l_1,l_2 \in {\mathbb {N}}_0$ such that $0 \le l_1, l_2 \le q$ we have

$$\begin{aligned} {\mathbb {E}}\bigg [&H_{2q-2l_1} \left( f(t) \right) H_{2q-2l_2} \left( f(s) \right) H_{2l_1} \left( \frac{f'(t)}{\sigma } \right) H_{2l_2} \left( \frac{f'(s)}{\sigma } \right) \bigg ]\\&= \sum _{n = \max (0, 2l_1 + 2l_2 - 2q)}^{\min (2l_1, 2l_2)} b_q(l_1, l_2, n) \left( \frac{r''(t-s)}{\sigma ^2} \right) ^n \left( \frac{r'(t-s)}{\sigma } \right) ^{2(l_1 + l_2 - n)} \\&\qquad \qquad \quad \qquad \qquad \quad \left( r(t- s) \right) ^{2(q-l_1-l_2)+n}. \end{aligned}$$

Before proving the lemma we first recall the diagram formula.

### Lemma 7

(The diagram formula [6, Page 432] [14, Theorem 1.36]) Let $X_1, \ldots , X_k$ be jointly Gaussian random variables, and $n_1, \ldots , n_k \in {\mathbb {N}}$. A Feynman diagram is a graph with $n_1 + \ldots + n_k$ vertices such that

- There are $n_i$ vertices labelled $X_i$ for each *i* (and each vertex has a single label). For a vertex *a* we write $X_{\ell (a)}$ for the label of *a*.
- Each vertex has degree 1.
- No edge joins 2 vertices with the same label.

Let ${\mathscr {D}}$ be the set of such diagrams. For $\gamma \in {\mathscr {D}}$ we define the value of $\gamma$ to be

$$\begin{aligned} v(\gamma ) = \prod _{(a,b) \in E(\gamma )} {\mathbb {E}}\left[ X_{\ell (a)} X_{\ell (b)} \right] \end{aligned}$$

where $E(\gamma )$ is the set of edges of $\gamma$. Then

$$\begin{aligned} {\mathbb {E}}\left[ H_{n_1}(X_1) \cdot \cdots \cdot H_{n_k} (X_k) \right] = \sum _{\gamma \in {\mathscr {D}}} v(\gamma ). \end{aligned}$$

### Proof of Lemma 6

We apply the diagram formula to the random variables $f(t),f(s),f'(t) / \sigma$ and $f'(s) / \sigma$ and corresponding integers $2(q-l_1), 2(q-l_2), 2l_1$ and $2l_2$ and denote by ${\mathscr {D}}$ the collection of relevant Feynman diagrams. Since ${\mathbb {E}}\left[ f(t) f'(t) \right] = {\mathbb {E}}\left[ f(s) f'(s) \right] = r'(0) = 0$, it is enough to consider diagrams whose edges do not join vertices labeled *f*(*t*) to $f'(t)/ \sigma$ or vertices labeled *f*(*s*) to $f'(s) / \sigma$.

**Fig. 1:**
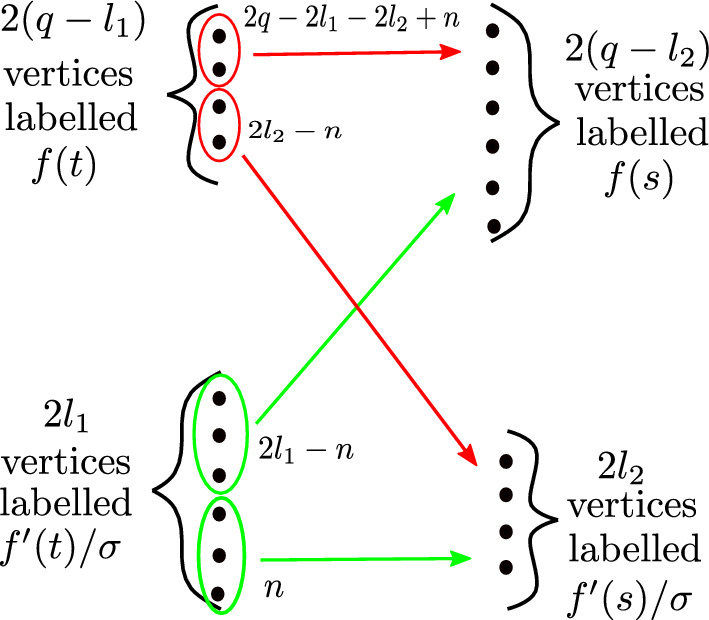
Counting the number of Feynman diagrams

Let *n* be the number of edges joining a vertex labeled $f'(t) / \sigma$ to a vertex labeled $f'(s) / \sigma$, see Fig. 1. Then $0 \le n \le \min (2l_1, 2l_2)$. Moreover, as the other vertices labeled $f'(t)/ \sigma$ must be joined to vertices labeled *f*(*s*), we see that $2l_1 - n \le 2q - 2l_2$, so $\max (0, 2l_1 + 2l_2 - 2q) \le n \le \min (2l_1, 2l_2)$. Further, every value of *n* in this range is attained by some diagram.

We compute the value of such a diagram to be

$$\begin{aligned} v(\gamma )&= {\mathbb {E}}\left[ f'(t)f'(s) / \sigma ^2 \right] ^{n} {\mathbb {E}}\left[ f'(t) f(s) / \sigma \right] ^{2l_1 - n}\\&\qquad {\mathbb {E}}\left[ f(t) f'(s) / \sigma \right] ^{2l_2-n} {\mathbb {E}}\left[ f(t) f(s) \right] ^{2q-2l_1-2l_2+n} \\&= \left( \frac{r''(t-s)}{\sigma ^2} \right) ^n \left( \frac{r'(t-s)}{\sigma } \right) ^{2(l_1 + l_2 - n)} \left( r(t-s)\right) ^{2(q-l_1-l_2)+n} . \end{aligned}$$

Finally, we count the number of such diagrams. There are

$$\begin{aligned} \left( {\begin{array}{c}2l_1\\ n\end{array}}\right) \left( {\begin{array}{c}2l_2\\ n\end{array}}\right) n! \end{aligned}$$

ways to choose *n* vertices labeled $f'(t) / \sigma$, to choose *n* vertices labeled $f'(s) / \sigma$ and to pair them. There are

$$\begin{aligned} \left( {\begin{array}{c}2q-2l_2\\ 2l_1-n\end{array}}\right) (2l_1-n)! \end{aligned}$$

ways to choose $2l_1-n$ vertices labeled *f*(*s*) and to pair them with the remaining vertices labeled $f'(t) / \sigma$. There are

$$\begin{aligned} \left( {\begin{array}{c}2q-2l_1\\ 2q-2l_1-2l_2+n\end{array}}\right) (2q-2l_1-2l_2+n)! \end{aligned}$$

ways to choose $2q-2l_1-2l_2+n$ vertices labeled *f*(*t*) and to pair them with the remaining ones labeled *f*(*s*). There are

$$\begin{aligned} (2l_2-n)! \end{aligned}$$

ways to pair the remaining vertices labeled *f*(*t*) and $f'(s) / \sigma$. Since these choices are independent, we multiply these counts to get that there are $b_q(l_1,l_2,n)$ such diagrams, where $b_q$ is given by (11). Applying the diagram formula completes the proof. $\square$

## Proofs of Theorem 1 (a) and (b)

In this section, we prove parts (a) and (b) of Theorem 1. Our method is to bound each $V_q(T)$ by $V_1(T)$ and apply Proposition 4. We achieve this by proving the following properties of the polynomials $P_q$ (recall (13)).

### Proposition 8

For all $q\ge 1$ we have $(x+z)^{2}\mid P_{q}(x,y,z)$.

### Proposition 9

Set $$M = \max (|x| + |y|, |y| + |z|)$$. Then

21 $$\begin{aligned} \frac{|P_q(x,y,z)|}{(x+z)^2} \le \frac{e^2}{\sqrt{\pi }} q^{3/2} 4^{q} M^{2q-2}. \end{aligned}$$

Proving Proposition 8 amounts to proving some identities for the coefficients of the polynomials $P_q$, which is deferred to Sect. 5 where we implement a general method due to Zeilberger [2]. We proceed to prove Proposition 9.

### Proof of Proposition 9

By Proposition 8, we may prove Proposition 9 by bounding the second derivative of $P_q$. To achieve this we borrow the main idea from the proof of Arcones’ Lemma [3, Lemma 1].

#### Proof of Proposition 9

Our goal is to bound $\frac{\partial ^2 P_q}{\partial x^2}$. For $k \le 2q-2$, define

$$\begin{aligned} \alpha _q(k) = {\left\{ \begin{array}{ll} 0, &{} \text {for odd } k, \\ \frac{1}{q!} \cdot \left( {\begin{array}{c}q\\ k/2\end{array}}\right) \frac{(2q-k)!k!}{k-1},&\text {for even }k, \end{array}\right. } \end{aligned}$$

which yields (recall (10))

$$\begin{aligned} \alpha _q(2k) = \left( {\begin{array}{c}q\\ k\end{array}}\right) \frac{(2q-2k)!(2k)!}{(2k-1)\cdot q!} = (2q-2k)!(2k)! \cdot a_q(k). \end{aligned}$$

Let $0\le k,l \le 2q-2$ and suppose that *n* is an integer such that $\max (0, l+k-2q+2) \le n \le \min (l,k)$. Recalling (11) we have

$$\begin{aligned} \alpha _q(2k) \alpha _q(2l)&= (2q-2k)! (2k)! (2q-2l)! (2l)! \cdot a_q(k) a_q(l) \\&= (2q-2k-2l+n)! (2k-n)!(2l-n)! n! \cdot a_q(k) a_q(l) b_q(k,l,n) \end{aligned}$$

and so

22 $$\begin{aligned}{} & {} a_q(k)a_q(l) b_q(k,l,n) \frac{\partial ^2 }{\partial x^2}\left[ x^{2(q-k-l)+n}\right] \nonumber \\{} & {} \quad = \frac{\alpha _q(2k) \alpha _q(2l) x^{2q-2k-2l-2+n}}{(2q-2k-2l-2+n)!(2k-n)!(2l-n)!n!}. \end{aligned}$$

Let

$$\begin{aligned} k_1 = 2q-2-k, \quad k_2 = k,\quad l_1 = 2q-2-l,\quad \text {and}\quad l_2 = l, \end{aligned}$$

define

$$\begin{aligned} \mathcal {A}(k,l)&= \left\{ \left( \begin{array}{cc} 2q-l-k-2+n &{} l-n \\ k-n &{} n \end{array} \right) : \max (0, l+k-2q+2) \le n \le \min (l,k) \right\} \\&= \left\{ a = \left( \begin{array}{cc} a_{11} &{} a_{12} \\ a_{21} &{} a_{22} \end{array} \right) : a_{ij} \in {\mathbb {N}}_{0}, a_{i1} + a_{i2} = k_{i}, a_{1i} + a_{2i} = l_{i} \right\} \end{aligned}$$

and

$$\begin{aligned} \tilde{\mathcal {A}}(k)=\bigcup _{l=0}^{2q-2} \mathcal {A}(k,l) = \left\{ a = \left( \begin{array}{cc} a_{11} &{} a_{12} \\ a_{21} &{} a_{22} \end{array} \right) : a_{ij} \in {\mathbb {N}}_{0}, a_{i1} + a_{i2} = k_{i} \right\} . \end{aligned}$$

Then, using (22) and recalling (12), we have

$$\begin{aligned} \frac{\partial ^2 {\widetilde{P}}_q}{\partial x^2}&= \sum _{k,l=0}^{q-1} \alpha _q(2k) \alpha _q(2l) \sum _{n=\max (0,2k+2l-2q+2)}^{\min (2k,2l)}\\&\qquad \frac{x^{2(q-k-l-1)+n}}{(2q-2k-2l-2+n)!} \frac{y^{2k-n}}{(2k-n)!} \frac{y^{2l-n}}{(2l-n)!} \frac{z^{n}}{n!} \\&= \sum _{k,l=0}^{q-1} \alpha _q(2k) \alpha _q(2l) \sum _{A \in \mathcal {A}(2k,2l)} \prod _{i,j=1}^{2} \frac{x_{ij}^{a_{ij}}}{a_{ij}!}\\&= \sum _{k,l=0}^{2q-2} \alpha _q(k) \alpha _q(l) \sum _{A \in \mathcal {A}(k,l)} \prod _{i,j=1}^{2} \frac{x_{ij}^{a_{ij}}}{a_{ij}!} \end{aligned}$$

where

$$\begin{aligned} x_{11} = x, \quad x_{12} = x_{21} = y, \quad \text {and}\quad x_{22} = z. \end{aligned}$$

We now bound

$$\begin{aligned} \left| \frac{\partial ^2 {\widetilde{P}}_q}{\partial x^2} \right|&\le \sum _{k,l=0}^{2q-2} \left| \alpha _q(k) \alpha _q(l) \right| \sum _{a \in \mathcal {A}(k,l)} \prod _{i,j=1}^{2} \frac{|x_{ij}|^{a_{ij}}}{a_{ij}!} \\&\le \sum _{k,l=0}^{2q-2} \left( \frac{\alpha _q(k)^2 + \alpha _q(l)^2}{2} \right) \sum _{a \in \mathcal {A}(k,l)} \prod _{i,j=1}^{2} \frac{|x_{ij}|^{a_{ij}}}{a_{ij}!} \end{aligned}$$

Algebraic manipulation of this last quantity yields

$$\begin{aligned} \left| \frac{\partial ^2 {\widetilde{P}}_q}{\partial x^2} \right| \le \sum _{k=0}^{2q-2} \alpha _q(k)^2 \sum _{l=0}^{2q-2} \sum _{a \in \mathcal {A}(k,l)} \prod _{i,j=1}^{2} \frac{|x_{ij}|^{a_{ij}}}{a_{ij}!} = \sum _{k=0}^{2q-2} \alpha _q(k)^2 \sum _{a \in \tilde{\mathcal {A}}(k)} \prod _{i,j=1}^{2} \frac{|x_{ij}|^{a_{ij}}}{a_{ij}!} \end{aligned}$$

Applying the Binomial Theorem to the last term gives

$$\begin{aligned}&\sum _{k=0}^{2q-2} \alpha _q(k)^2 \prod _{i=1}^{2} \frac{(|x_{i1}| + |x_{i2}|)^{k_i}}{k_i!} \le M^{2q-2} \sum _{k=0}^{2q-2} \frac{\alpha _q(k)^2}{k! (2q-2-k)!}\\&\le 4q^2 M^{2q-2} \sum _{k=0}^{2q-2} \frac{\alpha _q(k)^2}{k! (2q-k)!}\\&= 4q^2 M^{2q-2} \sum _{k=0}^{q-1} \frac{1}{(q!)^2} {\left( {\begin{array}{c}q\\ k\end{array}}\right) }^2 \frac{(2k)!(2q-2k)!}{(2k-1)^2} = 4q^2 c_q M^{2q-2}. \end{aligned}$$

where the last identity is due to Lemma 10 below, and we remind the reader of (14). We also have, from (13), that

$$\begin{aligned} \frac{\partial ^2 P_q}{\partial x^2} = \frac{\partial ^2 {\widetilde{P}}_q}{\partial x^2} + (2q-1)(2q-2)c_q \left( x^{2q-3}z + (2q-3)x^{2q-4}y^2 \right) . \end{aligned}$$

We next bound this final summand. Note that for $q=1$ this term vanishes. Otherwise, on the domain $D_M = \{ |x| + |y| \le M, |y| + |z| \le M \}$, it attains its maximum on the boundary, and a calculation reveals the maximum is attained at $|z| = |x| = M, y = 0$. Therefore

$$\begin{aligned} | x^{2q-3}z + (2q-3)x^{2q-4}y^2| \le M^{2q-2}. \end{aligned}$$

Combining these two estimates we obtain

$$\begin{aligned} \left| \frac{\partial ^2 P_q}{\partial x^2} \right| \le \left( 4q^2 + (2q-1)(2q-2) \right) c_q M^{2q-2}\le 8q^2 c_q M^{2q-2}. \end{aligned}$$

Using Sterling’s bounds^10^ we see that $$\left( {\begin{array}{c}2q\\ q\end{array}}\right) \ge \frac{2\sqrt{\pi }}{e^2}\frac{2^{2q}}{\sqrt{q}}$$ which yields

$$\begin{aligned} c_q = \frac{2^{4q}}{2q \left( {\begin{array}{c}2q\\ q\end{array}}\right) } \le \frac{e^2}{4\sqrt{\pi }}\frac{4^{q}}{\sqrt{q}} \end{aligned}$$

so that

23 $$\begin{aligned} \sup _{D_M} \left| \frac{\partial ^2 P_q}{\partial x^2} \right| \le \frac{2e^2}{\sqrt{\pi }}q^{3/2}4^{q} M^{2q-2}. \end{aligned}$$

By the mean value theorem,

$$\begin{aligned} P_q(x,y,z) = P_q(-z,y,z) + \frac{\partial P_q}{\partial x}(-z,y,z) (x+z) + \frac{1}{2} \frac{\partial ^2 P_q}{\partial x^2}(t,y,z) (x+z)^2 \end{aligned}$$

for some *t* between *x* and $-z$. It follows from Proposition 8 that $P_q(-z,y,z) = \frac{\partial P_q}{\partial x}(-z,y,z)=0$, so that

$$\begin{aligned} P_q(x,y,z)= \frac{1}{2} \frac{\partial ^2 P_q}{\partial x^2}(t,y,z) (x+z)^2. \end{aligned}$$

Note that $|t| \le \max (|x|, |z|) \le M - |y|$ and so by (23) we have

$$\begin{aligned} \frac{|P_q(x,y,z)|}{(x+z)^2} \le \frac{1}{2} \sup _{(t,y,z)\in D_M } \left| \frac{\partial ^2 P_q}{\partial x^2}(t,y,z) \right| \le \frac{e^2}{\sqrt{\pi }} q^{3/2} 4^{q} M^{2q-2}. \end{aligned}$$

$$\square $$

In the course of the proof we used the following computation.

#### Lemma 10

For all $q \in {\mathbb {N}}$ we have

$$\begin{aligned} c_q = \sum _{l=0}^{q} \left( {\begin{array}{c}2l\\ l\end{array}}\right) \left( {\begin{array}{c}2q-2l\\ q-l\end{array}}\right) \frac{1}{(2l-1)^2}. \end{aligned}$$

#### Proof

For $q \ge 0$, let us denote $$T_q = \sum _{l=0}^{q} \left( {\begin{array}{c}2\,l\\ l\end{array}}\right) \left( {\begin{array}{c}2q-2\,l\\ q-l\end{array}}\right) \frac{1}{(2\,l-1)^2}$$. Notice that

24 $$\begin{aligned} \sum _{q=0}^{\infty } T_q x^{2q} = \phi (x) \psi (x) \end{aligned}$$

where

$$\begin{aligned} \phi (x) = \sum _{l=0}^{\infty } \left( {\begin{array}{c}2l\\ l\end{array}}\right) \frac{x^{2l}}{(2l-1)^2}, \quad \text {and}\quad \psi (x) = \sum _{l=0}^{\infty } \left( {\begin{array}{c}2l\\ l\end{array}}\right) x^{2l} = \frac{1}{\sqrt{1-4x^2}}. \end{aligned}$$

We next compute $\phi$. We have

$$\begin{aligned} \frac{d}{dx} \left[ \frac{\phi (x)}{x} \right] = \sum _{l=0}^{\infty } \left( {\begin{array}{c}2l\\ l\end{array}}\right) \frac{x^{2l-2}}{2l-1} = -\frac{1}{x^2} \sqrt{1-4x^2}= \frac{d}{dx} \left[ \frac{\sqrt{1-4x^2}}{x} + 2\arcsin (2x) \right] \end{aligned}$$

and so $\frac{\phi (x)}{x} = \frac{\sqrt{1-4x^2}}{x} + 2\arcsin (2x) + C$ for some constant *C*. Since all the functions in this equation are odd, it follows that $C = 0$, and so $\phi (x) = \sqrt{1-4x^2} + 2x\arcsin (2x)$. Therefore, using the Taylor series (20) once more,

$$\begin{aligned} \phi (x)\psi (x)&= 1 + \frac{2x \arcsin (2x)}{\sqrt{1-4x^2}}\\&= 1 + \frac{x}{2} \frac{d}{dx} \left( \arcsin (2x) \right) ^2\\&= 1 + \frac{x}{2} \frac{d}{dx} \sum _{q=1}^{\infty } \frac{(4x)^{2q}}{2q^2 \left( {\begin{array}{c}2q\\ q\end{array}}\right) } = 1 + \sum _{q=1}^{\infty } \frac{4^{2q} x^{2q}}{2q \left( {\begin{array}{c}2q\\ q\end{array}}\right) }. \end{aligned}$$

Comparing this with (24) we conclude that $$T_q = \frac{2^{4q}}{2q \left( {\begin{array}{c}2q\\ q\end{array}}\right) }=c_q$$ for $q \ge 1$. $\square$

### Lower bound

In this subsection we show that the lower bound in Theorem 1 (b) actually holds for any process. We will also use this lower bound in deducing Theorem  1 (a) from Proposition 9. We note that the estimate (25) also appears in [22].

#### Proposition 11

For any SGP,

$$\begin{aligned} {{\,\textrm{Var}\,}}[N(T)] \ge \frac{\sigma ^2}{\pi ^2} T\int _{0}^T \left( 1-\frac{t}{T}\right) \left( r(t)+\frac{r''(t)}{\sigma ^2}\right) ^2 \ dt. \end{aligned}$$

In particular, for any non-degenerate SGP there exists a constant $C=C(\rho )>0$ such that

25 $$\begin{aligned} {{\,\textrm{Var}\,}}[N(T)] \ge C T, \quad \forall T>0. \end{aligned}$$

#### Proof

From Proposition 4 we have

$$\begin{aligned} {{\,\textrm{Var}\,}}[N(T)]\ge \frac{\sigma ^2}{4\pi ^2} V_1(T) \end{aligned}$$

and the first statement of Proposition 11 follows simply by computing

$$\begin{aligned} P_1(x,y,z) = 2(x+z)^2 \end{aligned}$$

which gives

26 $$\begin{aligned} V_1(T) = 4T\int _{0}^T \left( 1-\frac{t}{T}\right) \left( r(t)+\frac{r''(t)}{\sigma ^2}\right) ^2 dt. \end{aligned}$$

To deduce the second statement it is enough to find an interval *I* such that $\left| r+\frac{r''}{\sigma ^2} \right| \ge C>0$ on *I*. But this follows from the fact that $r''$ is continuous and *r* is not cosine. $\square$

### Proof of Theorem 1 (a)

Having Proposition 9 at our disposal, we are ready to prove Theorem 1 (a). Let

$$\begin{aligned} M' = \limsup _{|t| \rightarrow \infty } \varphi (t)<1 \end{aligned}$$

and choose $M \in \left( M', 1 \right)$. Then there exists some $T_0 > 0$ such that $\varphi (t) \le M$ for all $|t| > T_0$. We can rearrange (15) to obtain

27 $$\begin{aligned} V_q(T)\nonumber = V_q(T_0)&+ 2\left( T - T_0 \right) \int _{0}^{T_0} P_q \left( r(t), \frac{r'(t)}{\sigma }, \frac{r''(t)}{\sigma ^2} \right) \ dt \\&\quad + 2\int _{T_0}^T \left( T - t \right) P_q \left( r(t), \frac{r'(t)}{\sigma }, \frac{r''(t)}{\sigma ^2} \right) \ dt. \end{aligned}$$

Proposition 9 yields

28 $$\begin{aligned}&\left| \int _{T_0}^{T} \left( T - t \right) P_q \left( r(t), \frac{r'(t)}{\sigma }, \frac{r''(t)}{\sigma ^2} \right) \ dt\right| \le \frac{e^2}{\sqrt{\pi }} q^{3/2} 4^{q} M^{2q-2} \nonumber \\&\qquad \qquad \quad \qquad \int _{T_0}^{T} \left( T - t \right) \left( r(t) + \frac{r''(t)}{\sigma ^2} \right) ^2 \ dt \nonumber \\&\le \frac{e^2}{\sqrt{\pi }} q^{3/2} 4^{q} M^{2q-2} \int _{0}^{T} \left( T - t \right) \left( r(t) + \frac{r''(t)}{\sigma ^2} \right) ^2 \ dt \nonumber \\&= \frac{e^2}{\sqrt{\pi }} q^{3/2} 4^{q-1} M^{2q-2} V_1(T), \end{aligned}$$

see (26). Since $M<1$ we see that

$$\begin{aligned} \sum _{q=1}^\infty \frac{1}{4^q}\left| \int _{T_0}^{T} \left( T - t \right) P_q \left( r(t), \frac{r'(t)}{\sigma }, \frac{r''(t)}{\sigma ^2} \right) \ dt\right| <\infty . \end{aligned}$$

By Proposition 4, since we are assuming the Geman condition, we have $\sum _{q=1}^{\infty } \frac{V_q(T)}{4^q} < \infty$ for every $T>0$ and so we may write, from (27)

$$\begin{aligned} \sum _{q=1}^{\infty } \frac{V_q(T)}{4^q}&= \sum _{q=1}^{\infty } \frac{V_q(T_0)}{4^q} + \left( T - T_0 \right) \sum _{q=1}^{\infty } \frac{1}{4^q} \int _{0}^{T_0} P_q \left( r(t), \frac{r'(t)}{\sigma }, \frac{r''(t)}{\sigma ^2} \right) \ dt \\&\quad + \sum _{q=1}^{\infty } \frac{1}{4^q} \int _{T_0}^{T} \left( T - t \right) P_q \left( r(t), \frac{r'(t)}{\sigma }, \frac{r''(t)}{\sigma ^2} \right) \ dt. \end{aligned}$$

Combining this with (28) we get

$$\begin{aligned} \sum _{q=1}^{\infty } \frac{V_q(T)}{4^q}\le C_0 + C_1 T + C_2 V_1(T) \end{aligned}$$

where $C_0,C_1$ and $C_2$ depend on $T_0$ and *M*. Recalling Proposition 4 we have

$$\begin{aligned} {{\,\textrm{Var}\,}}[N(T)] \le \frac{\sigma ^2}{\pi ^2} \sum _{q=1}^{\infty } \frac{V_q(T)}{4^q} + \frac{1}{4} \le C_3 V_1(T) \end{aligned}$$

where we have used Proposition 11 for the final bound.

### Proof of Theorem 1 (b)

By (26) we need to show that ${{\,\textrm{Var}\,}}[N(T)] \sim \frac{\sigma ^2}{4\pi ^2} V_1(T)$. The lower bound follows immediately from Proposition 11 and so we focus on the upper bound. We proceed as in the previous section, but estimate more carefully. By Proposition 4 we have

$$\begin{aligned} {{\,\textrm{Var}\,}}[N(T)] \le \frac{\sigma ^2}{4\pi ^2} V_1(T) + \frac{\sigma ^2}{\pi ^2} \sum _{q=2}^{\infty } \frac{V_q(T)}{4^q} +\frac{1}{4}. \end{aligned}$$

Now fix $\varepsilon >0$ and choose $T_0 = T_0(\varepsilon )$ such that $\varphi (t) < \varepsilon$ for all $t>T_0$. As in the previous section we write

$$\begin{aligned} \sum _{q=2}^{\infty } \frac{V_q(T)}{4^q}&= \sum _{q=2}^{\infty } \frac{V_q(T_0)}{4^q} + \left( T - T_0 \right) \sum _{q=2}^{\infty } \frac{1}{4^q} \int _{0}^{T_0} P_q \left( r(t), \frac{r'(t)}{\sigma }, \frac{r''(t)}{\sigma ^2} \right) \ dt \\&\quad + \sum _{q=2}^{\infty } \frac{1}{4^q} \int _{T_0}^{T} \left( T - t \right) P_q \left( r(t), \frac{r'(t)}{\sigma }, \frac{r''(t)}{\sigma ^2} \right) \ dt \end{aligned}$$

and estimate

$$\begin{aligned} \left| \int _{T_0}^{T} \left( T - t \right) P_q \left( r(t), \frac{r'(t)}{\sigma }, \frac{r''(t)}{\sigma ^2} \right) \ dt\right| \le \frac{e^2}{2\sqrt{\pi }} q^{3/2} 4^{q} \varepsilon ^{2q-2} V_1(T). \end{aligned}$$

This yields

$$\begin{aligned} \sum _{q=2}^{\infty } \frac{V_q(T)}{4^q} =C_0 + C_1 T + \frac{2e^2}{\sqrt{\pi }} \sum _{q=2}^{\infty } q^{3/2} (4\varepsilon ^2)^{q-1} V_1(T)\le C_0 + C_1 T + C_3 \varepsilon ^2 V_1(T) \end{aligned}$$

and we finally note that since $r+\frac{r''}{\sigma ^2} \not \in \mathcal {L}^2(\mathbb {R})$ we have

$$\begin{aligned} \frac{V_1(T)}{T}\rightarrow \infty \end{aligned}$$

as $T\rightarrow \infty$. This completes the proof.

### Conjectural bounds

In this section we give some evidence in favor of the conjectures stated in the Introduction. The precise expression for the variance appearing in Proposition 4 establishes a way to prove even tighter upper bounds, by reducing to combinatorial statements about the polynomials $P_q$, defined in (13). It is not difficult to see that the vector $(r(t),r'(t)/\sigma ,r''(t)/\sigma ^2)$ always lies in the domain

$$\begin{aligned} D = \{(x,y,z) \in \mathbb {R}^3: x^2 + y^2 \le 1, y^2 + z^2 \le 1 \}. \end{aligned}$$

By Proposition 8, $R_q(x,y,z) = P_q(x,y,z)/(x+z)^2$ is a homogeneous polynomial and since *D* contains all segments to the origin, it follows that $R_q$ attains the maximum of its absolute value on the boundary. We expect that the maximum should be obtained at the points where $|x|=|z|$.

When $x = -z$, the same techniques employed in this paper show the value to be

$$\begin{aligned} \frac{P_q(x,y,z)}{(x+z)^2} \Big \vert _{z=-x} = 2^{2q-1} (x^2 + y^2)^{q-1} \end{aligned}$$

and so on this boundary component the value of $R_q$ is $2^{2q-1}$. We believe that this bound is the one relevant to Gaussian processes, however numerical computations suggest that $R_q$ can be much larger at the points where $x=z$. We believe that there is some ‘hidden’ structure that prevents *r*(*t*) from being close to $r''(t)/\sigma ^2$ in certain subregions of *D*. For example, if *r*(*t*) is close to 1 then we should be close to a local maximum and so we would expect $r''(t)$ to be negative. Understanding the ‘true domain’ where the vector $(r(t),r'(t)/\sigma ,r''(t)/\sigma ^2)$ ‘lives’ already appears to be a quite interesting question.

## Atomic spectral measure

### The proofs of Theorem 1 (c) and Corollary 3

In this section we consider the effect of atoms in the spectral measure, that is, we prove Theorem 1 (c) and Corollary 3. Our proof relies on the following proposition.

#### Proposition 12

Let $\mu$ be a signed-measure with $\int _\mathbb {R}d|\mu |<\infty$. Then $\mu$ contains an atom if and only if there exists $c>0$ such that

$$\begin{aligned} \int _{-T}^T (T-|t|) \, |{\widehat{\mu }}(t)|^2 dt \ge c T^2 \end{aligned}$$

for all $$T>0$$.

We postpone the proof of Proposition 12 to Sect. 4.2. We will also need the following result.

#### Lemma 13

Let *f* be a SGP with covariance kernel *r*, spectral measure $\rho$ and suppose that $\rho$ has a continuous component. Let $\psi (t) = A \cos (\sigma t + \alpha )$, where $A \in \mathbb {R}$, $\alpha \in [0,2\pi ]$ and $\sigma ^2 = -r''(0)$. Denote by $N_J(\psi ) = \#\{ t \in [0,\pi J/\sigma ]: f(t) = \psi (t) \}$ the number of crossings of the curve $\psi$ by the process. Then ${\mathbb {E}}[N_J(\psi )] = J$.

#### Proof

Denote the Gaussian density function by $\varphi$ and by $\Phi$ the corresponding distribution function. The generalised Rice formula [7, Equation 13.2.1] gives

$$\begin{aligned} {\mathbb {E}}N_J(\psi )&= \sigma \int _{0}^{\frac{\pi J}{\sigma }} \varphi (\psi (y)) \left[ 2 \varphi \left( \frac{\psi '(y)}{\sigma } \right) + \frac{\psi '(y)}{\sigma } \left( 2 \Phi \left( \frac{\psi '(y)}{\sigma }\right) - 1 \right) \right] dy \\&= \sigma \int _{0}^{\frac{\pi J}{\sigma }} \frac{e^{-\frac{A^2}{2} \cos ^2 (\sigma y+ \alpha )}}{\sqrt{2 \pi }} \left[ \frac{2e^{-\frac{A^2}{2} \sin ^2 (\sigma y + \alpha )}}{\sqrt{2 \pi }} \right. \\&\left. \qquad \qquad \quad - A \sin (\sigma y+ \alpha ) \left( 2 \Phi \left( -A \sin (\sigma y+ \alpha ) \right) - 1 \right) \right] dy \\&= J e^{-\frac{A^2}{2}} - \frac{\sigma }{\sqrt{2\pi }} \int _{0}^{\frac{\pi J}{\sigma }} e^{-\frac{A^2}{2} \cos ^2 (\sigma y+ \alpha )} \\&\qquad \qquad \quad A \sin (\sigma y+ \alpha ) \left( 2 \Phi \left( -A \sin (\sigma y+ \alpha ) \right) - 1 \right) dy\\&= J e^{-\frac{A^2}{2}} - \frac{\sigma }{\sqrt{2\pi }} \int _{0}^{\frac{\pi J}{\sigma }} e^{-\frac{A^2}{2} \cos ^2 (\sigma y+ \alpha )} |A| \sin (\sigma y+ \alpha ) \\&\qquad \qquad \quad \left( 2 \Phi \left( -|A| \sin (\sigma y+ \alpha ) \right) - 1 \right) dy. \end{aligned}$$

Write

$$\begin{aligned} F(y) = e^{-\frac{A^2}{2} \cos ^2 (y)} |A| \sin (y) \left( 2 \Phi \left( -|A| \sin (y) \right) - 1 \right) \end{aligned}$$

and notice that *F* is periodic with period $\pi$. This yields

29 $$\begin{aligned} {\mathbb {E}}N_J(\psi )= & {} J\left( e^{-\frac{A^2}{2}} - \frac{\sigma }{\sqrt{2\pi }} \int _{0}^{\frac{\pi }{\sigma }} F(\sigma y + \alpha ) dy \right) \nonumber \\= & {} J \left( e^{-\frac{A^2}{2}} - \frac{1}{\sqrt{2\pi }} \int _{0}^{\pi } F( y ) dy \right) . \end{aligned}$$

Moreover, since *F* is even we have

$$\begin{aligned} \int _{0}^{\pi } F(y) \ dy= & {} \int _{0}^{\frac{\pi }{2}} F(y) \ dy + \int _{\frac{\pi }{2 }}^{\pi } F(y) \ dy = \int _{0}^{\frac{\pi }{2 }} F(y) \ dy + \int _{-\frac{\pi }{2 }}^{0} F(y) \ dy \\= & {} 2 \int _{0}^{\frac{\pi }{2 }} F(y) \ dy. \end{aligned}$$

Substituting $u = |A| \cos (y)$ we obtain

$$\begin{aligned} - \frac{1}{\sqrt{2\pi }} \int _{0}^{\pi } F(y) dy&= - {\sqrt{\frac{2}{\pi }}} \int _{0}^{\pi /2} F(y) dy\\&= -{\sqrt{\frac{2}{\pi }}} \int _{0}^{|A|} e^{-\frac{u^2}{2}} \cdot \left( 2 \Phi \left( -\sqrt{A^2 - u^2} \right) - 1 \right) du \\&= \frac{2}{ \pi } \int _{0}^{|A|} \int _{0}^{\sqrt{A^2 - u^2}} e^{-\frac{u^2+v^2}{2}} \ dv \ du \\&= \frac{2}{ \pi } \int _{0}^{|A|} \int _{0}^{\pi /2} e^{-\frac{r^2}{2}} r d\theta dr = 1 - e^{-\frac{A^2}{2}}. \end{aligned}$$

Inserting this value into (29) yields the result. $\square$

#### Proof of Theorem 1 (c)

First we note that, by stationarity, ${{\,\textrm{Var}\,}}[N(T)]\le C T^2$ for some $C>0$. Assume that $\rho$ has an atom at a point different from $\sigma$. By (16) and (26), to show that ${{\,\textrm{Var}\,}}[N(T)]\ge \frac{c\sigma ^2}{2\pi ^2}T^2$ for some $c>0$ it is enough to see that

$$\begin{aligned} \int _{-T}^T \left( T-|t|\right) \left( r(t) + \frac{r''(t)}{\sigma ^2}\right) ^2 dt \ge c T^2. \end{aligned}$$

But this follows from Proposition 12 if we define the signed measure $\mu$ by $d\mu (\lambda ) = (1-\frac{\lambda ^2}{\sigma ^2})d\rho (\lambda )$ and notice that ${\hat{\mu }}=r+\frac{r''}{\sigma ^2}$ and that $\mu$ has an atom.

For the converse, notice that it is enough to check that for integer *J* we have

$$\begin{aligned} \frac{{{\,\textrm{Var}\,}}[N(\frac{\pi }{\sigma }J)]}{J^2}\rightarrow 0 \quad \text { as }\quad J\rightarrow \infty , \end{aligned}$$

since this implies that ${{\,\textrm{Var}\,}}[N(T)]=o(T^2)$, by stationarity. Assume first that $\rho$ has no atoms; we adapt the proof of [5, Thm 4]. By the Fomin-Grenander-Maruyama theorem, *f* is an ergodic process (see, e.g., [12, Sec. 5.10]). By standard arguments, this also implies that the sequence

$$\begin{aligned} \mathcal {N}_j=\# \left\{ t\in \Big [(j-1)\frac{\pi }{\sigma },j\frac{\pi }{\sigma }\Big ): \, f(t)=0\right\} . \end{aligned}$$

is ergodic. Recall that we assume the Geman condition, which implies that the first and second moments of

$$\begin{aligned} N\left( \frac{\pi }{\sigma }J\right) =\sum _{j=1}^J \mathcal {N}_j \end{aligned}$$

are finite. Thus, by von Neumann’s ergodic theorem, we have

$$\begin{aligned} \lim _{J\rightarrow \infty }\frac{N(\frac{\pi }{\sigma }J)}{J} = {\mathbb {E}}[\mathcal {N}_1]=1, \end{aligned}$$

where the convergence is both in $L^1$ and $L^2$ (see [31, Cor. 1.14.1]). We conclude that

$$\begin{aligned} \lim _{J\rightarrow \infty }\frac{{{\,\textrm{Var}\,}}[N(\frac{\pi }{\sigma }J)]}{J^2} =0. \end{aligned}$$

Finally suppose that $\rho = \theta \rho _c + (1-\theta ) \delta ^*_{\sigma }$ where $0<\theta <1$ and $\rho _c$ has no atoms. We may represent the corresponding process as

$$\begin{aligned} f(t)=\sqrt{\theta }f_c + \sqrt{(1-\theta )X}\cos (\sigma t + \Phi ) \end{aligned}$$

where $f_c$ is a SGP with spectral measure $\rho _c$, $X\sim \chi ^2(2)$, $\Phi \sim \text {Unif}([0,2\pi ])$, and moreover $f_c,X$ and $\Phi$ are pairwise independent. By the law of total variance and Lemma 13 we have

30 $$\begin{aligned} \nonumber {{\,\textrm{Var}\,}}\Big [N\Big (\frac{\pi }{\sigma }J\Big )\Big ]&={\mathbb {E}}\Big [{{\,\textrm{Var}\,}}\big [N\Big (\frac{\pi }{\sigma }J\Big )\Big |X,\Phi \big ]\Big ]+{{\,\textrm{Var}\,}}\Big [{\mathbb {E}}\big [N\Big (\frac{\pi }{\sigma }J\Big )\Big |X,\Phi \big ]\Big ] \\&= {\mathbb {E}}\Big [{{\,\textrm{Var}\,}}\big [N\Big (\frac{\pi }{\sigma }J\Big )\Big |X,\Phi \big ]\Big ]. \end{aligned}$$

We define, for $A\in \mathbb {R}$ and $\alpha \in [0,2\pi ]$,

$$\begin{aligned} \mathcal {N}^{A,\alpha }_j=\# \left\{ t\in \Big [(j-1)\frac{\pi }{\sigma },j\frac{\pi }{\sigma }\Big ): \, f_c(t)=A\cos (\sigma t+\alpha )\right\} . \end{aligned}$$

As before the process $f_c$ is ergodic, and so is the sequence $\mathcal {N}^{A,\alpha }_j$ for fixed *A* and $\alpha$. This implies that

$$\begin{aligned} \lim _{J\rightarrow \infty }\frac{{{\,\textrm{Var}\,}}[N(\frac{\pi }{\sigma }J)|X,\Phi ]}{J^2} =0 \end{aligned}$$

(almost surely), exactly as before. Furthermore, using stationarity we have

$$\begin{aligned} \frac{1}{J^2}{{\,\textrm{Var}\,}}\big [N\Big (\frac{\pi }{\sigma }J\Big )\Big |X,\Phi \big ]\le {{\,\textrm{Var}\,}}\big [N\Big (\frac{\pi }{\sigma }\Big )\Big |X,\Phi \big ] \end{aligned}$$

and using (30) we see that

$$\begin{aligned} {\mathbb {E}}\Big [{{\,\textrm{Var}\,}}\big [N\Big (\frac{\pi }{\sigma }\Big )\Big |X,\Phi \big ]\Big ]={{\,\textrm{Var}\,}}\big [N\Big (\frac{\pi }{\sigma }\Big )\big ]<+\infty , \end{aligned}$$

since we assume the Geman condition. It follows from dominated convergence that

$$\begin{aligned} \lim _{J\rightarrow \infty }\frac{1}{J^2} {\mathbb {E}}\Big [{{\,\textrm{Var}\,}}\big [N\Big (\frac{\pi }{\sigma }J\Big )\Big |X,\Phi \big ]\Big ] =0 \end{aligned}$$

whence $$ \lim _{J\rightarrow \infty }\frac{{{\,\textrm{Var}\,}}[N(\frac{\pi }{\sigma }J)]}{J^2} =0$$. $$\square $$

#### Remark

We remarked in the introduction that the presence of a special atom in Theorem 1 (c) is unique to the zero level; here we give a brief explanation. Indeed, consider the spectral measure $\rho = \theta \rho _0 + (1-\theta ) \delta ^*_{\alpha }$ where $0<\theta <1$, $\alpha \in \mathbb {R}$ and $\rho _0$ is a symmetric probability measure. We may represent the corresponding process as

$$\begin{aligned} f(t)=\sqrt{\theta }f_0 + \sqrt{(1-\theta )X}\cos (\alpha t + \Phi ) \end{aligned}$$

where $f_0$ is a SGP with spectral measure $\rho _0$, and *X* and $\Phi$ are as above. Denote by $N_\ell (T)$ the number of crossings of the level $\ell$ by the process *f*. Again using the law of total variance we have

$$\begin{aligned} {{\,\textrm{Var}\,}}\Big [N_\ell \Big (\frac{2\pi }{\alpha }J\Big )\Big ] \ge {{\,\textrm{Var}\,}}\Big [{\mathbb {E}}\big [N_\ell \Big (\frac{2\pi }{\alpha }J\Big )\Big |X,\Phi \big ]\Big ] \end{aligned}$$

and by stationarity and periodicity we have ${\mathbb {E}}\big [N_\ell \Big (\frac{2\pi }{\alpha }J \Big )\Big |X,\Phi \big ]=J{\mathbb {E}}\big [N_\ell \Big (\frac{2\pi }{\alpha }\Big )\Big |X,\Phi \big ]$. A necessary condition for the variance to be sub-quadratic is therefore that ${\mathbb {E}}\big [N_\ell \Big (\frac{2\pi }{\alpha }\Big )\Big |X,\Phi \big ]$ is deterministic, and one may check using Kac-Rice that this requires $\ell =0$ and $\alpha ^2=\int _\mathbb {R}\lambda ^2d\rho _0(\lambda )$. Specifically, one may check that the values at $X=0$ and as $X\rightarrow \infty$ cannot both equal ${\mathbb {E}}\Big [N_\ell \big (\frac{2\pi }{\alpha }\big )\Big ]$ unless these conditions are satisfied.

#### Proof of Corollary 3

Let $M = \limsup _{|t| \rightarrow \infty } \varphi (t)$, where $\varphi$ is defined in (3). By assumption we have $M < 1$ and we define

$$\begin{aligned} \theta _0 = \frac{1 - M}{\sqrt{2} - M}. \end{aligned}$$

We would like to apply Theorem 1 (a) to the spectral measure $\rho _\theta$. Writing $r_\theta = \mathcal {F}[\rho _\theta ]$ and $r = \mathcal {F}[\rho ]$ we have $r_\theta (t) = (1-\theta ) r(t) + \theta \cos (\sigma t)$, and $\sigma _\theta ^2 = -r_\theta ''(0) = \sigma ^2$. We accordingly compute

$$\begin{aligned} \varphi _{\theta }(t)&= \max \left\{ |r_\theta (t)| +\frac{|r'_\theta (t)|}{\sigma }, \, \frac{|r''_\theta (t)|}{\sigma ^2} + \frac{|r'_\theta (t)|}{\sigma } \right\} \\&\le \max \Big \{ (1-\theta )\left( |r(t)| +\frac{|r'(t)|}{\sigma }\right) + \theta (|\cos \sigma t|+|\sin \sigma t|), \\&\qquad \qquad \qquad (1-\theta )\left( \frac{|r''(t)|}{\sigma ^2} + \frac{|r'(t)|}{\sigma }\right) + \theta (|\cos \sigma t|+|\sin \sigma t|)\Big \} \\&\le (1-\theta )M + \theta \sqrt{2} \end{aligned}$$

and so

$$\begin{aligned} \limsup _{|t| \rightarrow \infty } \varphi _\theta (t) < 1 \end{aligned}$$

for $\theta <\theta _0$. Applying Theorem 1 (a) to $\rho _\theta$ and to $\rho$ we obtain

$$\begin{aligned} {{\,\textrm{Var}\,}}[N(\rho _\theta ; T) ]&\asymp T\int _{-T}^T \left( 1-\frac{|t|}{T}\right) \left( r_\theta (t)+\frac{r_\theta ''(t)}{\sigma _\theta ^2}\right) ^2 \ dt \\ {}&= (1-\theta )^2 T\int _{-T}^T \left( 1-\frac{|t|}{T}\right) \left( r(t)+\frac{r''(t)}{\sigma ^2}\right) ^2 \ dt \\&\asymp {{\,\textrm{Var}\,}}[N(\rho ;T)]. \end{aligned}$$

$$\square $$

### Proof of Proposition 12

We begin with a review of some elementary harmonic analysis that we will need, for more details and proofs see, e.g., Katznelson’s book [17, Ch. VI]. Let $\mathcal {M}(\mathbb {R})$ denote the space of all finite signed measures on $\mathbb {R}$ endowed with the *total mass* norm $\Vert \mu \Vert _1 = \int _\mathbb {R}d |\mu |$. Recall that the *convolution* of two measures $\mu , \nu \in \mathcal {M}(\mathbb {R})$ is given by $(\mu *\nu ) (E) = \int \mu (E-\lambda ) d\nu (\lambda )$ for any measurable set *E* and satisfies $\Vert \mu *\nu \Vert _1 \le \Vert \mu \Vert _1 \Vert \nu \Vert _1$ and $\mathcal {F}[\mu * \nu ] = \mathcal {F}[\mu ]\cdot \mathcal {F}[\nu ]$. Moreover, $\mathcal {F}[\cdot ]$ is a uniformly continuous map with $\Vert \mathcal {F}[\mu ]\Vert _\infty \le \Vert \mu \Vert _1$. We identify a function $f\in L^1$ with the measure whose density is *f*.

The following lemma is a version of Parseval’s identity, see [17, VI 2.2].

#### Lemma 14

(Parseval) If $f,\mathcal {F}[f]\in L^1(\mathbb {R})$ and $\nu \in \mathcal {M}(\mathbb {R})$, then $\int f d\nu =\frac{1}{2\pi }\int \mathcal {F}[f] \overline{\mathcal {F}[\nu ]}$.

A simple application of Parseval’s identity proves our next lemma.

#### Lemma 15

Suppose that $\mu ,\nu \in \mathcal {M}(\mathbb {R})$ and $S, \mathcal {F}[S]\in L^1(\mathbb {R})$. Then

$$\begin{aligned} \int (S *\mu ) d\nu = \frac{1}{2\pi } \int \mathcal {F}[S] \mathcal {F}[\mu ] \overline{\mathcal {F}[\nu ]}. \end{aligned}$$

#### Proof

Note that $S * \mu$ is a function and further that

$$\begin{aligned} \Vert S *\mu \Vert _1&\le \Vert \mu \Vert _1 \Vert S\Vert _1<\infty , \quad \text { and} \\ \Vert \mathcal {F}[S*\mu ] \Vert _1&= \Vert \mathcal {F}[S] \mathcal {F}[\mu ] \Vert _1 \le \Vert \mathcal {F}[\mu ]\Vert _\infty \Vert \mathcal {F}[S]\Vert _1 \le \Vert \mu \Vert _1 \Vert \mathcal {F}[S]\Vert _1 <\infty . \end{aligned}$$

A simple application of Lemma 14 finishes the proof. $\square$

We will also use the so-called ‘triangle function’

$$\begin{aligned} \mathcal {T}_T(t) = \left( 1-\frac{|t|}{T} \right) {\mathbbm {1}}_{[-T,T]}(t) \end{aligned}$$

which satisfies $\mathcal {T}_T = \mathcal {F}[\mathcal {S}_T]$ where^11^

$$\begin{aligned} \mathcal {S}_T(\lambda ) = \frac{T}{2\pi } \ {{\,\textrm{sinc}\,}}^2\left( \frac{T \lambda }{2}\right) . \end{aligned}$$

Notice that applying Lemma 15 to these functions, we obtain

$$\begin{aligned} \int _{-T}^T \left( 1-\frac{|t|}{T} \right) |{\widehat{\mu }}(t)|^2 dt = \int _\mathbb {R}\mathcal {T}_T |\mathcal {F}[\mu ]|^2 =2\pi \int (\mathcal {S}_T * \mu )\ d\mu , \end{aligned}$$

which is (6).

We are now ready to prove Proposition 12. First suppose that $\mu$ contains an atom at $\alpha$. Write $\mu = \mu _1+\mu _2$ where $\mu _1 = c\delta _\alpha$ for some $c\ne 0$ and $\mu _2(\{\alpha \})=0$. Note that

31 $$\begin{aligned} |\mu _2([\alpha -\varepsilon ,\alpha +\varepsilon ])|\le |\mu _2|([\alpha -\varepsilon ,\alpha +\varepsilon ])\downarrow 0, \text { as }\varepsilon \downarrow 0. \end{aligned}$$

We have

$$\begin{aligned} | \mathcal {F}[\mu ](t)|^2&= |\mathcal {F}[\mu _1](t)|^2 + 2\text {Re} \{\mathcal {F}[\mu _1](t)\overline{\mathcal {F}[\mu _2](t)}\} + |\mathcal {F}[\mu _2](t)|^2 \\ {}&\ge |c|^2 + 2\text {Re}\{ \mathcal {F}[\mu _1](t) \overline{\mathcal {F}[\mu _2](t)}\} \end{aligned}$$

Using this and Lemma 15 we obtain

$$\begin{aligned} \int _{-T}^{T} (T-|t|) |{\widehat{\mu }}(t)|^2 dt&= T \int _\mathbb {R}\mathcal {T}_T |\mathcal {F}[\mu ]|^2 \\&\ge |c|^2 T \int _\mathbb {R}\mathcal {T}_T + 2T \text {Re}\left\{ \int _\mathbb {R}\mathcal {T}_T \mathcal {F}[\mu _1] \overline{\mathcal {F}[\mu _2]} \right\} \\&= |c|^2 T^2 + 4\pi T \, \text {Re} \left\{ \int _\mathbb {R}\mathcal {S}_T * \mu _1 \ d\mu _2\right\} . \end{aligned}$$

It is therefore enough to show that $\int _\mathbb {R}(\mathcal {S}_T*\mu _1)\ d\mu _2 = o(T)$. We bound

$$\begin{aligned} \left| \int (\mathcal {S}_T*\mu _1)(\lambda ) \ d\mu _2(\lambda )\right|= & {} \left| \frac{cT}{2\pi }\int _\mathbb {R}{{\,\textrm{sinc}\,}}^2\big (\tfrac{T}{2}(\lambda -\alpha )\big ) \ d\mu _2(\lambda )\right| \\\le & {} \frac{|c|T}{2\pi } \int _\mathbb {R}{{\,\textrm{sinc}\,}}^2\big (\tfrac{T}{2}(\lambda -\alpha )\big ) \ d|\mu _2|(\lambda ). \end{aligned}$$

Let $I_\alpha (T) = \big [\alpha - \frac{\log T}{T},\alpha +\frac{\log T}{T}\big ]$. By (31) we have

$$\begin{aligned} \int _{I_\alpha (T)} {{\,\textrm{sinc}\,}}^2 \big (\tfrac{T}{2}(\lambda -\alpha )\big ) d|\mu _2|(\lambda ) \le |\mu _2|(I_\alpha (T))\rightarrow 0, \quad \text {as } T\rightarrow \infty . \end{aligned}$$

On $\mathbb {R}{\setminus } I_\alpha (T)$ we have $\frac{T}{2}|\lambda -\alpha |\ge \frac{\log T}{2}$, so that

$$\begin{aligned} \int _{\mathbb {R}\setminus I_\alpha (T)} {{\,\textrm{sinc}\,}}^2 \big (\tfrac{T}{2}(\lambda -\alpha )\big ) d|\mu _2|(\lambda ) \le \frac{4}{(\log T)^2} |\mu _2|(\mathbb {R}) \rightarrow 0, \quad \text {as } T\rightarrow \infty . \end{aligned}$$

This concludes the first part of the proof.

Conversely, suppose that $\mu$ contains no atoms. Recall that

$$\begin{aligned} \int _{-T}^T \left( 1-\frac{|t|}{T} \right) |{\widehat{\mu }}(t)|^2 dt = 2\pi \int (\mathcal {S}_T * \mu )\ d\mu . \end{aligned}$$

We will show that $|(\mathcal {S}_T*\mu )(\lambda )|= o(T)$, uniformly in $\lambda$, which will conclude the proof. As before, denoting $I_\lambda (T) = \big [\lambda - \frac{\log T}{T},\lambda +\frac{\log T}{T}\big ]$ we have

$$\begin{aligned} |( \mathcal {S}_T*\mu )(\lambda )| = \left| \int _\mathbb {R}\frac{T}{2\pi } {{\,\textrm{sinc}\,}}^2\big (\tfrac{T}{2}(\lambda -\tau \big ) d\mu (\tau ) \right| \le \frac{T}{2\pi } \left( |\mu |(I_\lambda (T)) + \frac{4 |\mu |(\mathbb {R})}{ (\log T)^2} \right) . \end{aligned}$$

It therefore suffices to prove the following claim.

#### Claim 16

Let $\nu$ be a non-negative, finite measure on $\mathbb {R}$ that contains no atoms. Then

$$\begin{aligned} \sup _{x\in \mathbb {R}} \nu \big ([x-\varepsilon ,x+\varepsilon ]\big ) \rightarrow 0, \quad \text {as } \varepsilon \downarrow 0. \end{aligned}$$

#### Proof

Denote $B(x, \varepsilon ) =[x-\varepsilon ,x+\varepsilon ]$ and $m(\varepsilon ) = \sup _{x\in \mathbb {R}} \nu \big (B(x, \varepsilon )\big )$. It is clear that $m(\varepsilon )$ decreases with $\varepsilon$ so $m(\varepsilon )$ must converge as $\varepsilon \downarrow 0$ to some non-negative limit, $2\delta \ge 0$. Suppose that $\delta >0$ and choose $N>0$ such that $\nu (\mathbb {R}{\setminus } [-N/2,N/2])<\delta$. Fix $n\in {\mathbb {N}}$ and divide $[-N,N]$ into disjoint ‘dyadic’ intervals

$$\begin{aligned} D_n = \left\{ [kN2^{-n},(k+1) N2^{-n} ): k\in {\mathbb {Z}}\cap [-2^n, 2^n) \right\} . \end{aligned}$$

For any $x\in \mathbb {R}$, either $B(x,\frac{N}{2^n})\subseteq \mathbb {R}{\setminus } [-N/2,N/2]$, which implies that $\nu (B(x,\frac{N}{2^n}))<\delta$, or $B(x,\frac{N}{2^n})\subseteq I \cup I'$ for some $I, I' \in D_{n-1}$. Therefore,

$$\begin{aligned} m\big (\tfrac{N}{2^n}\big ) \le \max \left( \delta , 2\sup _{I\in D_{n-1}} \nu (I) \right) . \end{aligned}$$

Recall that by definition of $\delta$ we have $m\big (\tfrac{N}{2^n}\big )\ge 2\delta$. We conclude that for every $n \in {\mathbb {N}}$ we can find $I_n \in D_n$ such that

32 $$\begin{aligned} \nu (I_{n}) \ge \delta . \end{aligned}$$

Next we shall construct a sequence of nested dyadic intervals $\{J_n\}_{n=0}^\infty$ such that, for all *n*,

$$\begin{aligned} J_n \in D_n, \quad J_{n+1}\subseteq J_{n}, \quad \nu (J_n) \ge \delta . \end{aligned}$$

This will imply, by Cantor’s lemma, that $\bigcap _{n} J_n = \{x\}$ for some $x\in \mathbb {R}$, and further that $\nu (\{x\}) = \lim _{n\rightarrow \infty }\nu (J_n) \ge \delta >0$. This contradicts the assumption that $\nu$ has no atoms, which will end our proof.

We start by setting $J_0 = [-N,N]$. Suppose that we have constructed $J_0\supset J_1 \supset J_2 \supset \dots \supset J_m$ such that for every $n>m$ we can find $I_n' \in D_n$ that satisfies

33 $$\begin{aligned} I_n'\subset J_m, \quad \text {and}\quad \nu (I_n') \ge \delta ; \end{aligned}$$

that is, the interval $J_m$ has a descendant of any generation whose $\nu$-measure is at least $\delta$. Notice that this holds for $m=0$ by (32). Notice that if (33) fails for both descendants of $J_m$ in the generation $D_{m+1}$, then it also fails for $J_m$, since $\nu (J)\ge \nu (J')$ for every descendant $J'\subseteq J$. This completes the inductive construction of $J_m$ and consequently the proof. $\square$

## Proof of Proposition 8

### Dehomogenisation

Our first step is based on the following lemma.

#### Lemma 17

Let *P*(*x*, *y*, *z*) be a homogeneous polynomial. Then $(x+z)^{2}\mid P(x,y,z)$ if and only if $P(-1,y,1)=0$ and $\frac{\partial P}{\partial x}(-1,y,1)=0$.

#### Proof

Consider the polynomial $f(x,y)=P(x,y,1)$ and write *f* as a polynomial in $x+1$ to obtain $f(x,y)=\sum _{j=0}^{d}a_{j}(y)\cdot (x+1)^{j}$. Suppose first that

34 $$\begin{aligned} a_{0}(y)=f(-1,y)=P(-1,y,1)=0 \end{aligned}$$

and

35 $$\begin{aligned} a_{1}(y)=\frac{\partial f}{\partial x}(-1,y)=\frac{\partial P}{\partial x}(-1,y,1)=0. \end{aligned}$$

It follows that $(x+1)^{2}\mid f(x,y)$, and we write $f(x,y)=(x+1)^{2}g(x,y)$.

As *P*(*x*, *y*, *z*) is homogeneous, one has

$$\begin{aligned} P(x,y,z)&=z^{\deg P}P\left( \frac{x}{z},\frac{y}{z},1\right) =z^{\deg P}f\left( \frac{x}{z},\frac{y}{z}\right) \\&=z^{\deg P}\left( \frac{x}{z}+1\right) ^{2}g\left( \frac{x}{z},\frac{y}{z}\right) =(x+z)^{2}\cdot z^{\deg P-2}g\left( \frac{x}{z},\frac{y}{z}\right) . \end{aligned}$$

Finally $z^{\deg P-2}g\left( \frac{x}{z},\frac{y}{z}\right)$ is a homogeneous polynomial, and we are done.

For the converse, note that if $(x+z)^{2}\mid P(x,y,z)$, then $(x+1)^{2}\mid f(x,y)$, hence equations (34) and (35) hold. $\square$

In light of Lemma 17, Proposition 8 is equivalent to the next proposition.

#### Proposition 18

For all $q \ge 1$ we have

- $$P_{q}(-1,y,1)=0$$, and
- $$\frac{\partial P_{q}}{\partial x}(-1,y,1)=0$$.

We shall therefore concentrate on proving Proposition 18.

### Reduction to a combinatorial identity

For $z\in {\mathbb {R}}$ and $k\in {\mathbb {Z}}$, we use the standard notation $(z)_{k}$ for the rising factorial Pochhammer symbol

$$\begin{aligned} (z)_{k}=z(z+1)\cdot \cdots \cdot (z+k-1)=\frac{\Gamma (z+k)}{\Gamma (z)} \end{aligned}$$

where the second equality holds for *z* not a non-positive integer. We next reformulate Proposition 18 in terms of the purely hypergeometric terms

$$\begin{aligned} H_q(l_{1},l_{2},k)=\frac{(-1)^{l_1+l_2}\left( -\frac{1}{2}\right) _{l_1} \cdot \left( -\frac{1}{2}\right) _{l_2}\cdot \left( \frac{1}{2}\right) _{q-l_{1}}\cdot \left( \frac{1}{2}\right) _{q-l_{2}}}{(2q-l_{1}-l_{2}-k)!(l_{2}-l_{1}+k)!(l_{1}-l_{2}+k)!(l_{1}+l_{2}-k)!} \end{aligned}$$

and

$$\begin{aligned} H'_q(l_{1},l_{2},k)= (2q-l_1-l_2-k) H_q(l_1, l_2, k), \end{aligned}$$

in order to be able to apply Zeilberger’s algorithm in Sect. 5.3. We note that $H_q, H'_q$ are defined for every $k, l_1, l_2 \in {\mathbb {Z}}$, by expressing everything in terms of the Gamma function.

#### Proposition 19

For all $q\ge 1$ we have

- $$\begin{aligned} \sum _{l_{1},l_{2}}H_q(l_{1},l_{2},k)={\left\{ \begin{array}{ll} 0, &{}\quad \text {for }\,k\ge 2,\\ 2^{-4q}(2q-1)c_q, &{}\quad \text {for }\,k=1,\\ 2^{-4q}c_q, &{}\quad \text {for }\,k=0. \end{array}\right. } \end{aligned}$$
- $$\begin{aligned} \sum _{l_{1},l_{2}}H'_q(l_{1},l_{2},k)={\left\{ \begin{array}{ll} 0, &{}\quad \text {for }\,k\ge 2,\\ 2^{-4q}(2q-1)(2q-2)c_q, &{}\quad \text {for }\,k=1,\\ 2^{-4q}(2q-1)c_q, &{}\quad \text {for }\,k=0. \end{array}\right. } \end{aligned}$$

#### Proof that Proposition 19 is equivalent to Proposition 18

A rearrangement of the terms in (13) yields

36 $$\begin{aligned} P_{q}(-1,y,1)=\sum _{k=0}^{q}(-1)^kd_{q}(k)\cdot y^{2k}+c_{q}\left( (2q-1)y^{2}-1\right) , \end{aligned}$$

where

$$\begin{aligned} d_{q}(k)=\sum _{\begin{array}{c} k\le l_{1}+l_{2}\le 2q-k\\ |l_{1}-l_{2}|\le k \end{array}}a_{q}(l_{1})a_q(l_{2})b_{q}(l_{1},l_{2},l_{1}+l_{2}-k)\cdot (-1)^{l_{1}+l_{2}}. \end{aligned}$$

Similarly, one obtains

$$\begin{aligned} \frac{\partial P_{q}}{\partial x}(-1,y,1)=\sum _{k=0}^{q}(-1)^kd'_{q}(k)\cdot y^{2k}+c_{q}(2q-1)\left( 1-(2q-2)y^{2}\right) \end{aligned}$$

where

$$\begin{aligned} d'_{q}(k)=\sum _{\begin{array}{c} k\le l_{1}+l_{2}\le 2q-k\\ |l_{1}-l_{2}|\le k \end{array}}(2q-l_{1}-l_{2}-k)\cdot a_{q}(l_{1})a_q(l_{2})b_{q}(l_{1},l_{2},l_{1}+l_{2}-k)\cdot (-1)^{l_{1}+l_{2}-1}. \end{aligned}$$

It is therefore enough to prove that

$$\begin{aligned} (-1)^{l_1+l_2}a_{q}(l_{1})a_q(l_{2})b_{q}(l_{1},l_{2},l_{1}+l_{2}-k) = 2^{4q} H_q(l_1, l_2, k), \end{aligned}$$

which is easily verified by standard algebraic manipulations. $\square$

### Proof of Proposition 19 (a)

We will use the multivariate Zeilberger algorithm for multi-sum recurrences of hypergeometric terms (see [2] and [18, Chapters 6 and 7]). For convenience we write

$$\begin{aligned} S_q(k) = \sum _{l_{1},l_{2}}H_q(l_{1},l_{2},k)= 2^{-4q} d_q(k). \end{aligned}$$

First, we will handle the case where $k=q$.

#### Lemma 20

For all $q \ge 2$ we have $S_q(q) = 0$.

#### Proof

We have

$$\begin{aligned} d_q(q)&= \sum _{l=0}^{q} a_q(l) a_q(q-l) b_q(l,q-l,0) = \frac{1}{(q!)^2} \sum _{l=0}^q {\left( {\begin{array}{c}q\\ l\end{array}}\right) }^2 \cdot \frac{(2q-2l)!(2l)!}{(2l-1)(2q-2l-1)} \\&=\sum _{l=0}^q \left( {\begin{array}{c}2q-2l\\ q-l\end{array}}\right) \frac{1}{2q-2l-1} \cdot \left( {\begin{array}{c}2l\\ l\end{array}}\right) \frac{1}{2l-1}. \end{aligned}$$

We write $$ \phi (x) = \sum _{l=0}^{\infty } \left( {\begin{array}{c}2\,l\\ l\end{array}}\right) \frac{1}{2\,l-1} x^l = - \sqrt{1-4x}$$. Then $\sum _{q=0}^{\infty } d_q(q) x^q = \phi (x)^2 = 1-4x$, showing that $d_q(q) = 0$ for all $q \ge 2$, whence the claim. $\square$

Next, we prove a recurrence relation for $S_q(k)$.

#### Lemma 21

For all $q\ge 1$ and all $k \ne q+2$ we have

$$\begin{aligned}{} & {} \frac{q^{2}}{8(2k-2q-3)(k-q-2)}S_{q}(k)+\frac{4kq-4q^{2}+2k-7q-4}{4(2k-2q-3)(k-q-2)}S_{q+1}(k) +S_{q+2}(k)=0. \end{aligned}$$

#### Proof

Let us begin by defining some rational functions in 4 variables. Let

$$\begin{aligned} Q_q^{(1)}(l_1,l_2,k)&=4q^2 (l_1-l_2-k) +4 q k^3 +8qk^2(4-l_1-l_2)\\&+4q k \left( l_1-l_2\right) ^2 + 2q k(4l_2+14l_2 -11) +2q (2l_1 l_2 +3l_1-9l_2+3)\\&+4k(2k+1)(3-2l_1)(2l_2-1) +12l_1l_2-6l_1-18l_2+9,\\ Q_q^{(2)}(l_1,l_2,k)&= 8l_1^{2}k-4l_1^{2}q+4l_1l_2q-12l_1kq\\&+4l_1q^{2}-4l_2q^{2}+4kq^{2}-8l_1^{2}+4l_1l_2-12l_1k \\&+10l_1q-6l_2q+10kq+6l_1-2l_2+4k-2q-1 \end{aligned}$$

and

$$\begin{aligned} Q_q(l_1,l_2,k)=32k(2k-2q-3)(k-q-2)\cdot (2q-l_1-l_2-k+1)_4. \end{aligned}$$

Define also

$$\begin{aligned} R_q^{(1)}(l_1,l_2,k)=\frac{Q_q^{(1)}(l_1,l_2,k)(1/2+q-l_1)(l_1+l_2-k)(l_1-l_2+k)}{Q_q(l_1,l_2,k)} \end{aligned}$$

and

$$\begin{aligned} R_q^{(2)}(l_1,l_2,k)=-\frac{Q_q^{(2)}(,l_1,l_2,k)(1/2+q-l_2)(l_1+l_2-k)(l_2-l_1+k)}{Q_q(l_1,l_2,k)}. \end{aligned}$$

Applying Zeilberger’s algorithm yields the following identity of rational functions, which can be verified directly by expanding (and should be interpreted in the usual way at the poles):

$$\begin{aligned}&\frac{q^{2}}{8(2k-2q-3)(-q+k-2)}+\frac{4kq-4q^{2}+2k-7q-4}{4(2k-2q-3)(-q+k-2)}\cdot \frac{H_{q+1}(l_1,l_2,k)}{H_q(l_1,l_2,k)}\\&+\frac{H_{q+2}(l_1,l_2,k)}{H_q(l_1,l_2,k)}\\&=R_q^{(1)}(l_1+1,l_2,k)\cdot \frac{H_q(l_1+1,l_2,k)}{H_q(l_1,l_2,k)}-R_q^{(1)}(l_1,l_2,k)\\&\qquad +R_q^{(2)}(l_1,l_2+1,k)\cdot \frac{H_q(l_1,l_2+1,k)}{H_q(l_1,l_2,k)}-R_q^{(2)}(l_1,l_2,k). \end{aligned}$$

Therefore, after multiplying both sides by $H_q(l_1,l_2,k)$, one gets

$$\begin{aligned}&\frac{q^{2} H_q(l_1,l_2,k)}{8(2k-2q-3)(-q+k-2)} \\&+\frac{4kq-4q^{2}+2k-7q-4}{4(2k-2q-3)(-q+k-2)}H_{q+1}(l_1,l_2,k)+H_{q+2}(l_1,l_2,k)\\&=G_q^{(1)}(l_1+1,l_2,k)-G_q^{(1)}(l_1,l_2,k)+G_q^{(2)}(l_1,l_2+1,k)-G_q^{(2)}(l_1,l_2,k), \end{aligned}$$

where $G_q^{(1)}(l_1,l_2,k)=R_q^{(1)}(l_1,l_2,k)\cdot H_q(l_1,l_2,k)$, and $G_q^{(2)}(l_1,l_2,k)=R_q^{(2)}(l_1,l_2,k)\cdot H_q(l_1,l_2,k)$. Tedious but routine manipulations show that $G_q^{(1)}$ and $G_q^{(2)}$ are well-defined at the poles of $R_q^{(1)}$ and $R_q^{(2)}$. We can now sum over all $l_1,l_2$ on both sides, noting that $H_q$ (and therefore $G_q^{(1)}$ and $G_q^{(2)}$) vanish for $|l_1|$ or $|l_2|$ sufficiently large, and get

$$\begin{aligned} \frac{q^{2}}{8(2k-2q-3)(-q+k-2)}S_{q}(k)+\frac{4kq-4q^{2}+2k-7q-4}{4(2k-2q-3)(-q+k-2)}S_{q+1}(k)+S_{q+2}(k)=0, \end{aligned}$$

as claimed. $$\square $$

Now Proposition 19 easily follows from Lemma 21, by induction.

#### Proof of Proposition 19 (a)

We proceed by induction on *q*. For the base case note that

$$\begin{aligned} P_{1}(x,y,z)=2(x+z)^{2} \end{aligned}$$

whence, recalling (36) and the relation $S_q(k) = 2^{-4q} d_q(k)$,

$$\begin{aligned} \left( S_{1}(0)-\frac{4}{2^{4}}\right) +\left( \frac{4}{2^{4}}-S_{1}(1)\right) y^{2}+\sum _{k\ge 2}S_{1}(k)y^{2k}=\frac{1}{2^{4}}P_{1}(-1,y,1)=0. \end{aligned}$$

This implies that

$$\begin{aligned} S_{1}(k)={\left\{ \begin{array}{ll} 0, &{} \text {for }k\ge 2,\\ \frac{1}{4}, &{} \text {for }k=1,\\ \frac{1}{4}, &{} \text {for }k=0, \end{array}\right. } \end{aligned}$$

which is exactly the case $q=1$. Similarly, one verifies the formula for $q=2$.

Using now Lemma 21, it is clear that we have $S_{q+2}(k)=0$ for all $2\le k<q+2$. By Lemma 20, this also holds for $k=q+2$. By definition, $S_{q+2}(k)=0$ for $k > q+2$. It remains to consider the cases $k=0,1$. Assume that

$$\begin{aligned} \begin{array}{cc} S_{q}(0)=2^{-4q} c_q, &{}S_{q}(1)= 2^{-4q}(2q-1)c_q \\ S_{q+1}(0)=2^{-4(q+1)} c_{q+1}, &{}S_{q+1}(1)= 2^{-4(q+1)}(2q+1)c_{q+1}. \end{array} \end{aligned}$$

Then from Lemma 21 we have

$$\begin{aligned} -S_{q+2}(0)&=\frac{q^{2}}{8(2q+3)(q+2)}\cdot \frac{1}{2q\cdot \left( {\begin{array}{c}2q\\ q\end{array}}\right) }-\frac{4q^{2}+7q+4}{4(2q+3)(q+2)}\cdot \frac{1}{(2q+2)\cdot \left( {\begin{array}{c}2q+2\\ q+1\end{array}}\right) } \\&=-\frac{1}{2(q+2)\cdot \left( {\begin{array}{c}2q+4\\ q+2\end{array}}\right) } \end{aligned}$$

and similarly

$$\begin{aligned} -S_{q+2}(1)&=\frac{q^{2}}{8(2q+1)(q+1)}\cdot \frac{2q-1}{2q\cdot \left( {\begin{array}{c}2q\\ q\end{array}}\right) }-\frac{4q^{2}+3q+2}{4(2q+1)(q+1)}\cdot \frac{2q+1}{(2q+2)\cdot \left( {\begin{array}{c}2q+2\\ q+1\end{array}}\right) }\\&=-\frac{1}{4 \left( {\begin{array}{c}2q+2\\ q+1\end{array}}\right) } \end{aligned}$$

as claimed. $$\square $$

### Proof of Proposition 19 (b)

The development is very similar to that of the previous section, and we shall accordingly give less detail. We define

$$\begin{aligned} S'_q(k) = \sum _{l_{1},l_{2}}H'_q(l_{1},l_{2},k) \end{aligned}$$

and notice that $S'_q(k) = 2^{-4q} d'_q(k)$. We begin with a recurrence relation, similar to before.

#### Lemma 22

For all $q\ge 1$ and all $k \ne 2q-1$, we have

$$\begin{aligned} \frac{q(k-2q-1)}{2(2k-2q-1)(k-2q+1)}S'_{q}(k)+S'_{q+1}(k)=0. \end{aligned}$$

#### Proof

This time we define

$$\begin{aligned} Q'_{1}(q,l_1,l_2,k)&=2l_2k-4kq+4l_2q-k+2l_2-2q-1, \\ Q'_{2}(q,l_1,l_2,k)&=2k^{2}-2l_2k-4l_2q+3k-2l_2+2q+1 \end{aligned}$$

and

$$\begin{aligned} Q'(q,l_1,l_2,k)=4k(2k-2q-1)(k-2q+1)(2q-l_1-l_2-k)(2q-l_1-l_2-k+1). \end{aligned}$$

Define also

$$\begin{aligned} R'_{1}(q,l_1,l_2,k)=\frac{Q'_{1}(q,l_1,l_2,k)(1/2+q-l_1)(l_1+l_2-k)(l_1-l_2+k)}{Q^{'}(q,l_1,l_2,k)} \end{aligned}$$

and

$$\begin{aligned} R'_{2}(q,l_1,l_2,k)=\frac{Q'_{2}(q,l_1,l_2,k)(1/2+q-l_2)(l_1+l_2-k)(l_2-l_1+k)}{Q^{'}(q,l_1,l_2,k)}. \end{aligned}$$

Applying Zeilberger’s algorithm again yields

$$\begin{aligned}&\frac{q(k-2q-1)}{2(2k-2q-1)(k-2q+1)}H'_q(l_1,l_2,k)+H'_{q+1}(l_1,l_2,k) \\ {}&= G'_{1}(q,l_1+1,l_2,k)-G'_{1}(q,l_1,l_2,k)+G'_{2}(q,l_1,l_2+1,k)-G'_{2}(q,l_1,l_2,k) \end{aligned}$$

where $G'_{1}(q,l_1,l_2,k)=R'_{1}(q,l_1,l_2,k)\cdot H'_q(l_1,l_2,k)$, and $G'_{2}(q,l_1,l_2,k)=R'_{2}(q,l_1,l_2,k)\cdot H'_q(l_1,l_2,k)$. We can now sum over all $l_1,l_2$ on both sides and get the result. $\square$

Proposition 19 (b) now follows by induction, as before.

## Footnotes

1 To see this, note first that $r'$ and $r''$ are uniformly continuous. Now suppose that $r(t)\rightarrow 0$ but there exists an $\varepsilon >0$ and a sequence $\{t_n \}_{n=1}^{\infty }$ such that $|r'(t_n)| > 2 \varepsilon$ for all *n* and $t_n \rightarrow \infty$. By the uniform continuity of $r'$ we get $\left| r'(t) - r'(t_n) \right| < \varepsilon$ for $|t - t_n| < \delta$. Hence $|r'(t)| \ge |r'(t_n)| - |r'(t)-r'(t_n)| > \varepsilon$ and so

$$\begin{aligned} \left| \int _{t_n - \delta }^{t_n + \delta } r'(t) \ dt \right| = \int _{t_n-\delta }^{t_n+\delta } |r'(t)| dt \ge 2 \varepsilon \delta > 0. \end{aligned}$$

But, we can also compute

$$\begin{aligned} \lim _{n \rightarrow \infty } \left| \int _{t_n-\varepsilon }^{t_n+\varepsilon } r'(t) \ dt \right| = \lim _{n \rightarrow \infty } |r(t_n + \varepsilon ) - r(t_n-\varepsilon )| = 0, \end{aligned}$$

which is absurd. The same proof shows that $r''\rightarrow 0$.

2 While some of the results in [22] give information about atoms at points other than $\sigma$, our result is the first to show that atoms at $\sigma$ have a different effect on the variance, and are therefore special.

3 Notice that ${\mathbb {E}}[N(\rho _\theta ; T)]$ is independent of $\theta$.

4 This formula was based on the ideas of Kac–Rice, and indeed such a formula was known to physicists [27] and had been proved mathematically assuming the existence of $r^{(vi)}(0)$ (see the footnote on [30, Page 188]).

5 In fact we produce a family of such covariance functions.

6 Strictly speaking we first add a small computable quantity, which leads to the difference between $P_q$ and ${\widetilde{P}}_q$ in Sect. 2.

7 We adopt the standard convention $\frac{1}{n!}=0$ when *n* is a negative integer.

8 The conditions (1–3) in [21] are satisfied in our setting: (3) is trivial since $\psi \equiv 0$, (2) is precisely the Geman condition, and (1) is a consequence of the fact that *r* is twice differentiable and can be written as the cosine transform of the spectral measure.

9 Under the Geman condition, one cannot assume that *f* is continuously differentiable, and ‘conversely’ a continuously differentiable process need not satisfy the Geman condition, see [11, Section 4]. However the existence of $r''$ implies the existence of the derivative in quadratic mean of the process, and this is how the object $f'$ should be understood if the process is not differentiable.

10 The constants here are not asymptotically optimal, but this is irrelevant for our purposes.

11 We use the normalisation ${{\,\textrm{sinc}\,}}(x)=\frac{\sin x}{x}$.
